# Quantification of Prostate Cancer Metabolism Using 3D Multiecho bSSFP and Hyperpolarized [1‐^13^C] Pyruvate: Metabolism Differs Between Tumors of the Same Gleason Grade

**DOI:** 10.1002/jmri.28467

**Published:** 2022-10-31

**Authors:** Rafat Chowdhury, Christoph A. Mueller, Lorna Smith, Fiona Gong, Marianthi‐Vasiliki Papoutsaki, Harriet Rogers, Tom Syer, Saurabh Singh, Giorgio Brembilla, Adam Retter, Max Bullock, Lucy Caselton, Manju Mathew, Eoin Dineen, Thomas Parry, Jürgen Hennig, Dominik von Elverfeldt, Andreas B. Schmidt, Jan‐Bernd Hövener, Mark Emberton, David Atkinson, Alan Bainbridge, David G. Gadian, Shonit Punwani

**Affiliations:** ^1^ Centre for Medical Imaging, Division of Medicine University College London London UK; ^2^ Department of Radiology, Medical Physics, Medical Center University of Freiburg, Faculty of Medicine, University of Freiburg Freiburg Germany; ^3^ Division of Surgery and Interventional Science University College London London UK; ^4^ German Cancer Consortium (DKTK) partner site Freiburg and German Cancer Research Center (DKFZ) Heidelberg Germany; ^5^ Department of Radiology, and Neuroradiology, Section Biomedical Imaging, MOIN CC, University Medical Center Schleswig‐Holstein University of Kiel Kiel Germany; ^6^ Department of Medical Physics and Biomedical Engineering University College London Hospitals NHS Foundation Trust London UK; ^7^ UCL Great Ormond Street Institute of Child Health London UK; ^8^ Department of Radiology University College London Hospitals NHS Foundation Trust London UK

**Keywords:** hyperpolarized [1‐^13^C] pyruvate, metabolic ^13^C‐MRI, bSSFP

## Abstract

**Background:**

Three‐dimensional (3D) multiecho balanced steady‐state free precession (ME‐bSSFP) has previously been demonstrated in preclinical hyperpolarized (HP) ^13^C‐MRI in vivo experiments, and it may be suitable for clinical metabolic imaging of prostate cancer (PCa).

**Purpose:**

To validate a signal simulation framework for the use of sequence parameter optimization. To demonstrate the feasibility of ME‐bSSFP for HP ^13^C‐MRI in patients. To evaluate the metabolism in PCa measured by ME‐bSSFP.

**Study Type:**

Retrospective single‐center cohort study.

**Phantoms/Population:**

Phantoms containing aqueous solutions of [1‐^13^C] lactate (2.3 M) and [^13^C] urea (8 M). Eight patients (mean age 67 ± 6 years) with biopsy‐confirmed Gleason 3 + 4 (*n* = 7) and 4 + 3 (*n* = 1) PCa.

**Field Strength/Sequences:**

^1^H MRI at 3 T with T_2_‐weighted turbo spin‐echo sequence used for spatial localization and spoiled dual gradient‐echo sequence used for B_0_‐field measurement. ME‐bSSFP sequence for ^13^C MR spectroscopic imaging with retrospective multipoint IDEAL metabolite separation.

**Assessment:**

The primary endpoint was the analysis of pyruvate‐to‐lactate conversion in PCa and healthy prostate regions of interest (ROIs) using model‐free area under the curve (AUC) ratios and a one‐directional kinetic model (*k*_P_). The secondary objectives were to investigate the correlation between simulated and experimental ME‐bSSFP metabolite signals for HP ^13^C‐MRI parameter optimization.

**Statistical Tests:**

Pearson correlation coefficients with 95% confidence intervals and paired *t*‐tests. The level of statistical significance was set at *P* < 0.05.

**Results:**

Strong correlations between simulated and empirical ME‐bSSFP signals were found (*r* > 0.96). Therefore, the simulation framework was used for sequence optimization. Whole prostate metabolic HP ^13^C‐MRI, observing the conversion of pyruvate into lactate, with a temporal resolution of 6 seconds was demonstrated using ME‐bSSFP. Both assessed metrics resulted in significant differences between PCa (mean ± SD) (AUC = 0.33 ± 012, *k*_P_ = 0.038 ± 0.014) and healthy (AUC = 0.15 ± 0.10, *k*_P_ = 0.011 ± 0.007) ROIs.

**Data Conclusion:**

Metabolic HP ^13^C‐MRI in the prostate using ME‐bSSFP allows for differentiation between aggressive PCa and healthy tissue.

**Evidence Level:**

2

**Technical Efficacy:**

Stage 1

The incidence of prostate cancer (PCa) in the United Kingdom is over 48,000 cases per year and 40% of these cases are diagnosed at a late stage^1^ . The adoption of multiparametric MRI (mpMRI) for the prebiopsy detection of PCa has been a milestone in improving patient management.^2^ Compared to the prior approach of nontargeted transrectal ultrasound biopsy of the prostate in patients suspected of harboring PCa, the new mpMRI‐based targeted biopsy paradigm has increased the sensitivity for detecting disease from approximately 50%–90%.^3^ Moreover, around 30% of patients investigated for PCa are found to have a normal mpMRI and can now safely avoid an unnecessary biopsy.^3^

A remaining clinical challenge is to identify those individuals with histologically proven PCa, whose cancer would likely result in death if left untreated.^4^ Gleason grade assessment has traditionally been the method to discriminate the aggressiveness of disease.^5^ However, a large majority of patients are diagnosed with the same Gleason grade of 3 + 4; a heterogeneous group in which some patients show tumor progress, while others could safely avoid radical treatments and their side effects.^6^ An aggressive cancer is suggested to be more metabolically active than indolent forms,^7^ thus metabolic imaging may offer a potential approach for assessing disease aggressiveness.^7^, ^8^ The assessment of PCa metabolism could avoid the unnecessary treatment of patients with nonlethal disease.

Hyperpolarized ^13^C MRI (HP‐MRI) provides in vivo measurement of the conversion of organic molecules such as [1‐^13^C] pyruvate into their downstream metabolic products.^6^ HP‐MRI employs dissolution dynamic nuclear polarization (d‐DNP), a technique that can produce up to 10^5^‐fold enhancement of the spin polarization of ^13^C‐labeled nuclei, as compared to thermal equilibrium at 3 T.^9^ Hyperpolarization can be achieved through different methods, such as parahydrogen‐induced polarization (PHIP), spin‐exchange optical pumping (SeOP), and dissolution dynamic nuclear polarization (d‐DNP).^8^ In the context of clinical ^13^C‐MR, however, only d‐DNP has been approved for clinical studies. The corresponding increase in MR signal intensity has opened new possibilities for imaging metabolism. For example, following the injection of hyperpolarized [1‐^13^C] pyruvate, its conversion to [1‐^13^C] lactate can be monitored noninvasively in real time.^10^ In patients with cancer, such studies offer a window into investigations of the Warburg effect (the preferred cellular metabolism of pyruvate to lactate over aerobic respiration).^11^, ^12^ A limitation, however, of HP‐MRI is the short effective half‐life and irreversible decay of the signal from the hyperpolarized agent, with that of [1‐^13^C] pyruvate observed as ~30 seconds ex vivo.^13^, ^14^ Unlike in conventional MRI, the hyperpolarized signal does not recover; as a result, there is a need for specialized MR excitation and acquisition methods (pulse sequences) that provide the requisite combination of spatiotemporal and spectral resolution.

Several pulse sequences can be considered.^11^, ^15^ Nonlocalized spectroscopy provides high spectral and temporal resolution, but it yields only a single spectrum from a region that is defined by the sensitivity profile of the MR excitation and receiver coils.^6^ Chemical shift imaging (CSI) enables signals to be spatially resolved, but it has a relatively poor temporal resolution.^8^, ^11^ Echo‐planar spectroscopic imaging (EPSI) sequences afford improved temporal resolution but have limited spectral bandwidth, and they are particularly prone to errors caused by susceptibility gradients at air/tissue interfaces, such as those which occur with prostate imaging procedures.^16^ Instead of broadband or multiband excitation and sophisticated spectral‐spatial signal encoding, a metabolite‐specific excitation combined with a fast signal readout has also been shown to be a robust approach.^17^

Another possible approach employs the Dixon technique, invented for fat‐water separation in ^1^H‐MRI.^18^, ^19^ This approach involves the acquisition of echo images at different echo times (TEs), and it allows for the reconstruction of metabolite‐specific chemical shift maps, with the spectral resolution being limited by the number of acquired echo images and reconstruction parameters.^20^ If only a few downstream hyperpolarized metabolites are expected, and there is a priori knowledge of the chemical shifts, this technique allows immense reduction of spectral bandwidth and thus reduction of scan time.^21^

The balanced steady‐state free precession (bSSFP) sequence is widely used in anatomical MRI as it affords high spatiotemporal resolution and signal‐to‐noise ratio (SNR); many vendors are offering a rendition of the sequence (eg true fast imaging with steady‐state precession [TrueFISP, Siemens], fast imaging employing steady‐state acquisition [FIESTA, GE], and balanced fast‐field echo [Balanced‐FFE, Philips]). This sequence, adapted with a bipolar multiecho (ME) balanced gradient readout and Dixon type postprocessing, could provide an optimal balance of spectral sampling, spatial resolution, and overall acquisition time in HP‐MRI.^19^, ^22^, ^23^ Previous preclinical studies demonstrated this sequence in both phantom and animal subjects, successfully analyzing the conversion of hyperpolarized [1‐^13^C] pyruvate into [1‐^13^C] lactate.^23^, ^24^

This study aimed to investigate optimization and application of a three‐dimensional (3D) ME‐bSSFP sequence for metabolic, whole‐organ prostate imaging with hyperpolarized ^13^C‐MRI.

## Materials and Methods

Written informed consent was obtained for eight individuals with biopsy confirmed tumors (seven with Gleason 3 + 4 and one with a 4 + 3), who were then recruited to this study to evaluate whether ME‐bSSFP is a suitable means by which to quantify tumors, in conjunction with HP‐MRI (https://clinicaltrials.gov/ct2/show/NCT03687645).

All simulations and data processing were performed using MATLAB R2019a (MathWorks, Natick, MA, USA). Phantoms were prepared using ^13^C‐labeled materials obtained from Merck & Co. (Kenilworth, NJ, USA). All MRI‐based phantom and patient examinations were performed using a custom‐designed ^13^C clamshell transmit and dual tune ^1^H/^13^C receive‐only endorectal coil with dimensions: 97 × 31 × 22 mm (Rapid Biomedical GmbH, Rimpar, Germany), using a 3 T MRI (Siemens Biograph mMR, Siemens Healthineers, Erlangen, Germany). The endorectal coil houses a 1 mL, 8 M ^13^C‐urea phantom as reference and for power calibration (described in the Supplementary Material SI5). A custom [1‐^13^C] lactate (Merck & Co.) phantom (2 M, 2.3 mL, 0.23% w/v Sodium Azide and 0.15% w/w Dotarem) was used in this study for simulation validation. The gradient strengths for the Siemens Biograph mMR are as follows: MQ gradients (45 mT/m; 200 T/m/sec), as per the Siemens Healthineers manual.

### Pulse Sequence Parameter Optimization

**FIGURE 1:**
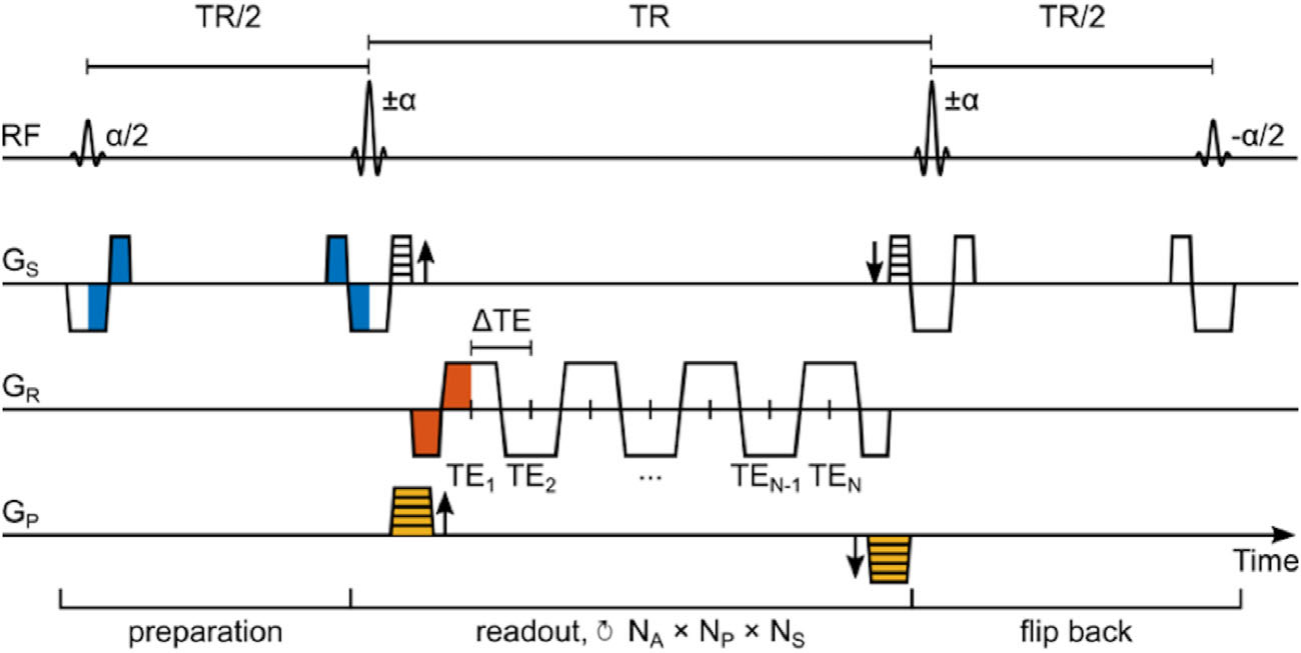
Pulse sequence diagram for a ME‐bSSFP sequence. A slab‐selective excitation of α/2 and dummy cycles of ±α refocusing pulses were used for magnetization preparation. The acquisition part is built of a bipolar gradient multiecho readout with *N* echoes separated by ΔTE and centered around TR/2. Repetitions of the readout over *N*_A_ averages and (*N*_P_ × *N*_S_) phase and slice encoding steps result in a total acquisition time of *N*_A_ × *N*_P_ × *N*_S_ × TR + 2 × (TR/2).

A 3D variant of a previously described ME‐bSSFP sequence (Fig. 1) was used in this study with a slab‐selective, sinc‐shaped excitation pulse of flip angle (FA) ɑ/2, followed by refocusing pulses of FA ɑ with alternating polarity (±).^23^ Between 5 and 10 dummy cycles at the beginning of the pulse sequence were used to create a transient transverse magnetization.^25^ A Cartesian center‐out *k*‐space readout trajectory was employed with (*N*_p_ × *N*_s_) phase and slice encoding steps, and a bipolar multigradient‐echo readout centered between refocusing pulses to acquire *N*_e_ consecutive echoes separated by a fixed difference in TE (ΔTE). An ɑ/2 flip‐back pulse, played out at the end of the sequence, transferred remaining transversal magnetization (M_T_) back into longitudinal magnetization after the end of each scan.^19^, ^23^

With steady‐state techniques such as ME‐bSSFP, the signal amplitude varies periodically as a function of relative resonance frequency. This signal variation can cause a failure of the sequence's refocusing mechanism and can result in banding artifacts.^26^ The artifacts can be mitigated by using a short repetition time (TR). Among other reasons, [1‐^13^C] pyruvate is an interesting molecule for hyperpolarization due to its rather long T_1_ and T_2_ relaxation constants, reported as 15–30 seconds and 0.9 seconds, respectively.^19^, ^23^ For such spin systems with T_1_ > > T_2_, the use of small FA lowers the pulse sequence‐induced consumption of the hyperpolarized magnetization by keeping the major fraction of the excited magnetization in longitudinal orientation where the dominant decay rate is T_1_.^25^ At the same time, FA must be high enough to achieve sufficient measurable transverse magnetization for good SNR. However, the use of high FA (>30°) and short TRs may be prohibited by the MRI system, to limit specific absorption rate (SAR) of energy in patients. All of these concerns suggested the need for careful selection of TR and FA to preserve signal lifetime, avoid high SAR, and afford a high SNR.^20^, ^26^, ^27^

A previously described numerical simulator was used to calculate theoretical bSSFP signals for various TRs: 8–22 msec and FA: 5°–35°.^23^ This was done to find optimal imaging parameters for on‐resonant lactate (Δ*f* = 0) and off‐resonant pyruvate (Δ*f* = −385 Hz). Simulator parameters: number of pulses = 64, TE_center_ = TR/2, T_1_ = 13.1 seconds, T_2_ = 0.6 seconds. These simulations were empirically validated using the [1‐^13^C] lactate phantom and the 3D ME‐bSSFP sequence on the 3 T MRI; field of view (FOV): 90 × 90 × 80 mm^3^, voxel size: 11.3 × 11.3 × 10 mm^3^, FA: 10°–30°, TR: 8–22 msec, number of echoes (NE): 7, number of signal averages (NA): 6. The simulator was subsequently used to calculate signal evolutions for a set of TR values and fixed FA hyperpolarized [1‐^13^C] pyruvate imaging: TR: 15.8/17.6/21.0 msec, FA: 24°, Δ*f*: −700 to 100 Hz, number of pulses = 64, TE_center_ = TR/2, T_1_ = 13.1 seconds, T_2_ = 0.6 seconds. Further information on the simulator and phantom experiments can be found in the supplementary information (SI1).

### Hyperpolarized [1‐^13^C] Pyruvate Preparation

[1‐^13^C] Pyruvic acid was mixed with AH111501 electron paramagnetic agent and loaded into a fluid path under aseptic conditions.^6^, ^11^ The fluid path was irradiated with microwaves at <1 K for approximately 2 h in a clinical hyperpolarizer (SPINLab, GE Healthcare, Chicago, Il, USA). Polarization level (*n* = 8), measured by the SpinLab, at the point of dissolution: 27 ± 4 (mean ± 95% CI). The frozen sample was then dissolved with sterile heated water (38 mL) and neutralized with 17.5 g sterile trometamol buffer (333 mM Tris and 600 mM NaOH); >40 mL of hyperpolarized [1‐^13^C] pyruvate solution was produced at the following concentration: 251–270 mM, temperature: 35.6–39°C, and pH: 6.7–7.6. Injection of the hyperpolarized agent started 70–81 seconds after dissolution, with data acquisition beginning immediately after the completion of the injection. Exactly 40 mL of hyperpolarized [1‐^13^C] pyruvate was injected into each patient and followed by a saline flush (60 mL).

### Clinical ^1^H Imaging/ ^13^C Imaging

Prior to injection of hyperpolarized [1‐^13^C] pyruvate, anatomical axial and sagittal T_2_‐weighted ^1^H (^1^H‐T_2_W) images were acquired with a turbo spin‐echo (TSE) sequence for tumor localization; FOV: 180 × 140 × 90 mm^3^, voxel size: 0.7 × 0.7 × 3 mm^3^, TR: 5400 msec, TE: 109 msec, slice thickness: 3 mm, number of slices: 30, echo train length (ETL): 15, NA: 1, FA: 90°. Additionally, a B_0_ field map was obtained for each patient via a dual gradient echo sequence; FOV: 360 × 360 × 80 mm^3^, voxel size: 1.4 × 1.4 × 10 mm^3^ FA: 15°, TR: 329 msec, TE_1_: 2.39 msec, TE_2_: 7.17 msec, ETL: 2, NA: 3. Calculation and analysis of the field maps are detailed in the Supplementary Material (SI2).

The ^13^C transmit and receive frequency were carefully adjusted on the lactate resonance frequency (Δ*f* = 0 Hz) by setting it +620 Hz from the peak resonance of the urea phantom within the endorectal coil. Dynamic HP‐MRI with the ME‐bSSFP sequence was initiated with completion of the hyperpolarized [1‐^13^C] pyruvate injection; FOV: 90 × 90 × 80 mm^3^, voxel size: 11.3 × 11.3 × 10 mm^3^, FA: 24°, TR: 15.8 msec, ΔTE: 1.1 msec, NE: 7, readout gradient bandwidth (BW_read_): 1200 Hz/px, NA: 6. Four consecutive ME‐bSSFP acquisitions were obtained directly after the injection, followed by the acquisition of a nonlocalized spectrum using a single repetition of a free induction decay (FID) sequence: FA: 10°, TR: 1000 msec, NA: 1. This was followed by a further 15 ME‐bSSFP acquisitions, an additional nonlocalized spectrum and another 15 ME‐bSSFP acquisitions. The interleaved spectra were used to identify frequencies for metabolite map reconstruction via iterative decomposition with echo asymmetry and least‐squares estimation (IDEAL). For further details on the metabolite separation, see Supplementary Material SI3.

### Data Analysis

The echo images obtained during ^13^C‐MRI were passed through an IDEAL model^20^, ^23^ using the calculated field map as an initial guess, with metabolite maps reconstructed at the frequencies identified in the non‐localized spectra. Further information on the IDEAL model used is provided in the Supplementary Material (SI4). The individual frequencies used in the reconstruction process are listed in the Supplementary Material (SI9). For the clinical dataset, quantification involved the drawing of regions of interest (ROI) at the biopsy confirmed tumor and a healthy area. The change in metabolite SNR, as a function of time within the ROIs was extracted. Tumor and normal ROIs were identified using multiparametric MRI: dynamic contrast‐enhanced imaging, diffusion‐weighted imaging, apparent diffusion coefficient maps, and T2‐weighted turbo‐spin echo images. Biopsy reports were also used to identify tumor ROIs while histology and biopsy reports were used to identify nonsuspicious (normal) areas of the prostate. The locations of the ROIs were verified by a radiologist (>10 years of experience).

For kinetic analysis of the metabolite signals, lactate‐to‐pyruvate area under the temporal curve (AUC) ratios were calculated^28^, ^29^; as well as a one‐directional kinetic model to derive the forward rate constant (*k*_P_) for the enzymatic conversion of pyruvate to lactate. Further information on the analysis methods is provided in the Supplementary Material (SI6).

### Statistical Analysis

The empirical and simulated signals from the numerical simulator work were normalized and compared in each instance with a Pearson correlation coefficient. A paired *t*‐test was also performed in each instance to check for statistically significant differences between simulation and experiment. All statistical analysis was performed using custom MATLAB scripts.

From the patient exams, the mean, the standard deviation, and 95% confidence interval values for healthy and tumor ROIs were then calculated for the kinetic metrics: AUC and *k*_P_. A Pearson correlation coefficient of 0.797, between AUC and *k*_P_ was found. A paired *t*‐test was performed to assess statistically significant differences between the healthy and tumorous tissues across all patients (*n* = 8). The level of statistical significance was set at *P* < 0.05.

## Results

### Numerical Simulator Work

**FIGURE 2:**
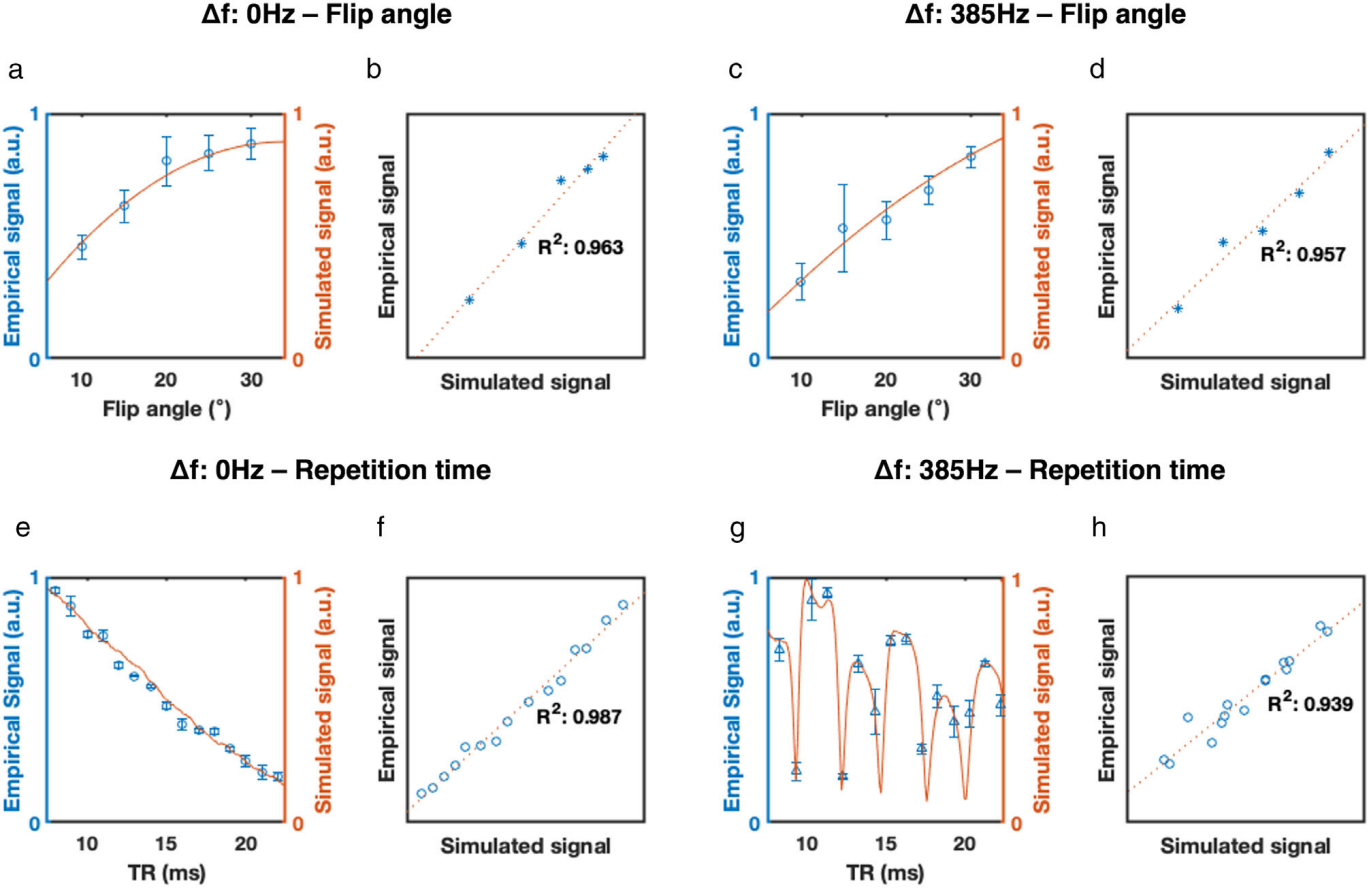
Simulated and empirical experimental results for testing of the ME‐bSSFP simulator: change in empirical and simulated signal as a function of flip angle at 0 Hz (a) and 385 Hz (c), at a constant repetition time (19.5 msec); change in empirical and simulated signal as a function of repetition time at 0 Hz (d) and 385 Hz (g) at a constant flip angle (30°). Correlation between resulting signal (simulated and empirical) as a function of changes in flip angle between empirical and simulated signals at 0 Hz (b) and 385 Hz (d). Correlation between resulting signal (simulated and empirical) as a function of changes in repetition time between empirical and simulated signals at 0 Hz (f) and 385 Hz (h).

The correlation coefficients between normalized empirical and simulated data for FA and TR at Δ*f* = 0 Hz and Δ*f* = 385 Hz were all found to be greater than 0.96 (Fig. 2). No statistically significant differences were found between the mean values of the normalized empirical and simulated data, across all testing experiments, affirming the simulator's ability to appropriately reflect the ME‐bSSFP sequence's empirical performance (Table 1).

**TABLE 1:** Correlation Between Empirical and Simulated Signals Using ME‐bSSFP at Δ*f* = 0 Hz and Δ*f* = −385 Hz

Δ*f* = 0 Hz		*N*	Mean	SD.	95% CI		*R* ^2^	Pearson	*P*
FA	Sim.	5	0.823	0.201	0.429	1.218	0.963	0.988	0.552
	Exp.	5	0.814	0.185	0.451	1.177			
TR	Sim.	12	0.805	0.111	0.587	1.023	0.987	0.994	0.279
	Exp.	12	0.751	0.151	0.454	1.048			

Δ*f* = −385 Hz		*N*	Mean	SD	95% CIs		*R* ^2^	Pearson	*P*
FA	Sim.	5	0.599	0.239	0.131	1.067	0.957	0.982	0.552
	Exp.	5	0.567	0.272	0.034	1.177			
TR	Sim.	12	0.716	0.172	0.378	1.054	0.939	0.969	0.279
	Exp.	12	0.595	0.254	0.097	1.092			

*N* refers to the number of measurements taken, while the mean, standard deviation, and 95% CI are also shown. The *R* ^2^ and Pearson correlation coefficient between the simulated and their respective experimental signals are shown, while the *P* values from paired *t*‐tests are included.

FA = flip angle; TR = repetition time; N = number of experiments; *P* = *P* value from paired *t*‐test.

### Pulse Sequence Parameter Optimization

**FIGURE 3:**
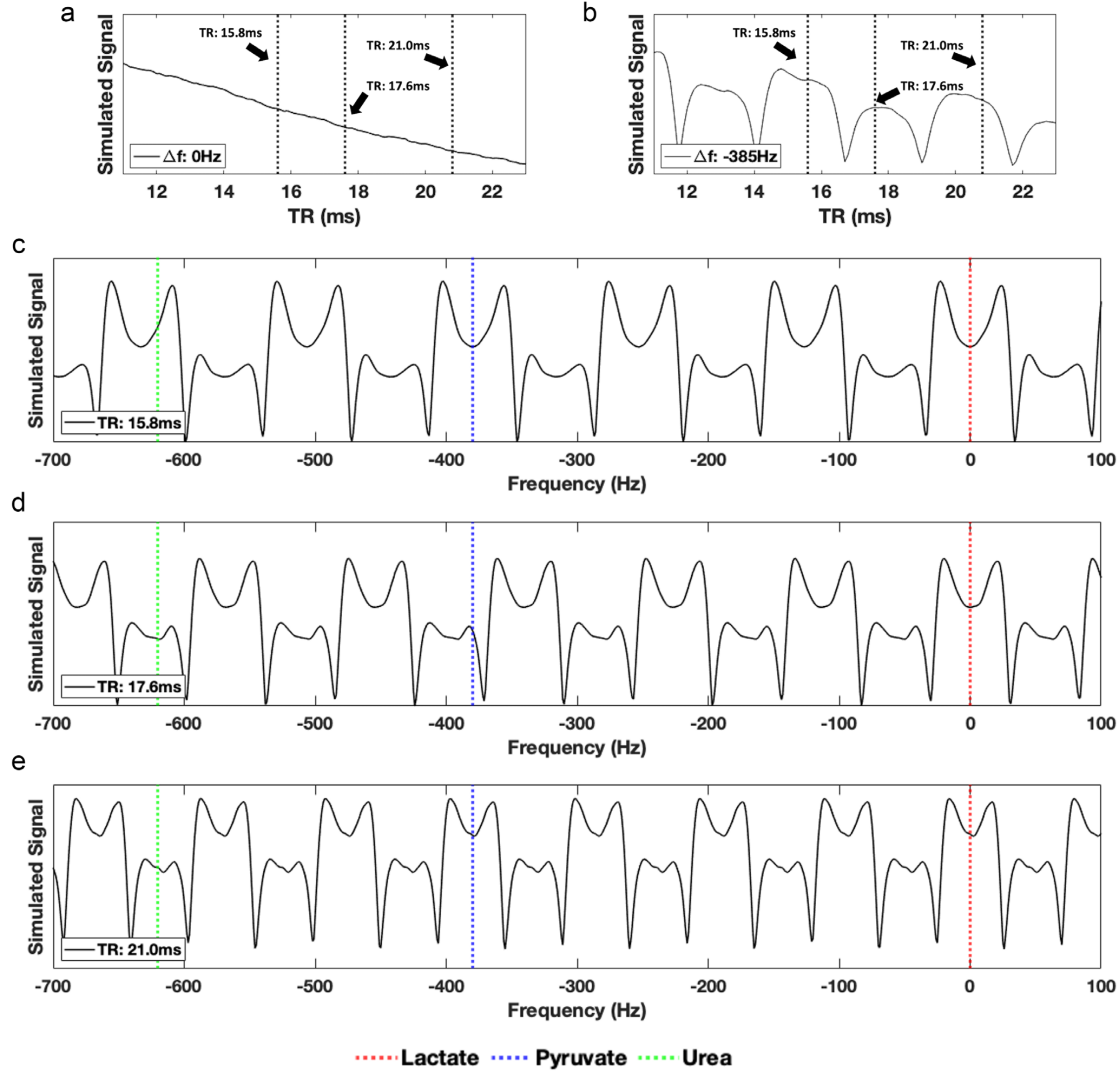
Change in simulated signal (transverse magnetization—M_T_) as a function of TR for a fixed flip angle (24°) at Δ*f* = 0 (a) and Δ*f* = −385 (b). On these graphs (a) and (b) the repetition times: 15.8, 17.6, and 21.0 msec are shown by dotted black lines. Further modeling was then performed using these TRs (c–e). Subsequently, simulated signal as a function of FA (24°), for a range of resonance frequencies (−700: 100 Hz) for specific TRs: 15.8 (c), 17.6 (d), and 21.0 msec (e) was modeled. In each instance, the expected resonance frequency of lactate (Δ*f* = 0, red), pyruvate (Δ*f* = −385, blue), and urea (Δ*f* = −620, green) is shown by dotted lines to indicate whether metabolites appear in pass or stop bands.

The maximum applicable FA in the sequence protocols was limited to 24° due to SAR. Modeling the bSSFP signal response for this FA at Δ*f* = 0 Hz and −385 Hz (Fig. 3a,b) yielded three potential TRs: 15.8, 17.6, and 21.0 msec. Further modeling demonstrated that the expected lactate frequency (Δ*f* = 0 Hz) consistently appeared in the middle of areas of high signal pass bands (Fig. 3c–e), while the respective pyruvate frequency (Δ*f* = −385 Hz) was not in the middle of a pass band (stop band) for TR = 17.6 msec (Fig. 3d) and as such this TR was considered suboptimal. Despite both TRs, 15.8 msec and 21.0 msec, being deemed suitable for use, TR = 15.8 msec improved the temporal resolution (~6 sec/dynamic scan) compared to 21 msec (~8 sec/dynamic scan).

### Application in Patients

**FIGURE 4:**
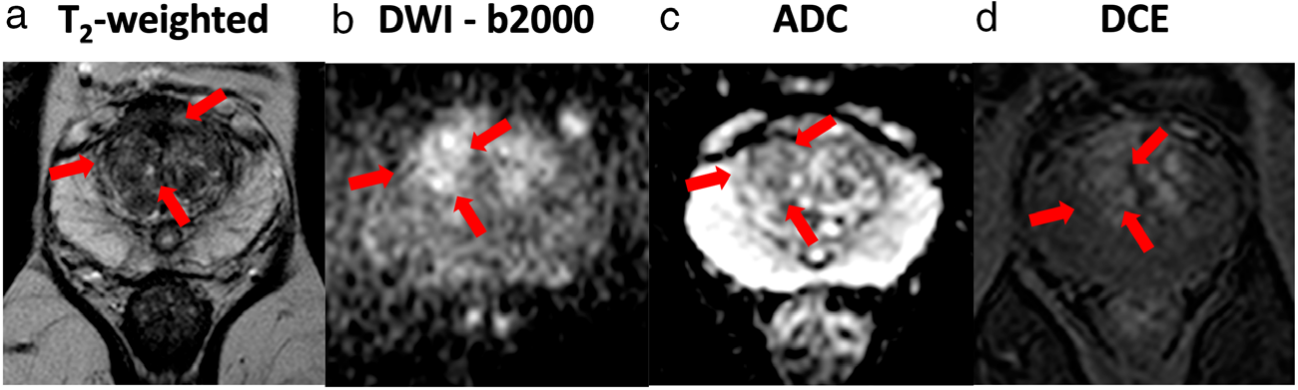
Example of the mpMRI data from a subject with PCa. (Left to right) (a) T2‐weighted image (T2W), (b) diffusion‐weighted Image (DWI)—b2000, (c) apparent diffusion coefficient (ADC) map and (d) dynamic contrast‐enhanced MRI (DCE). In each image, the tumor location is outlined with red arrows.

**FIGURE 5:**
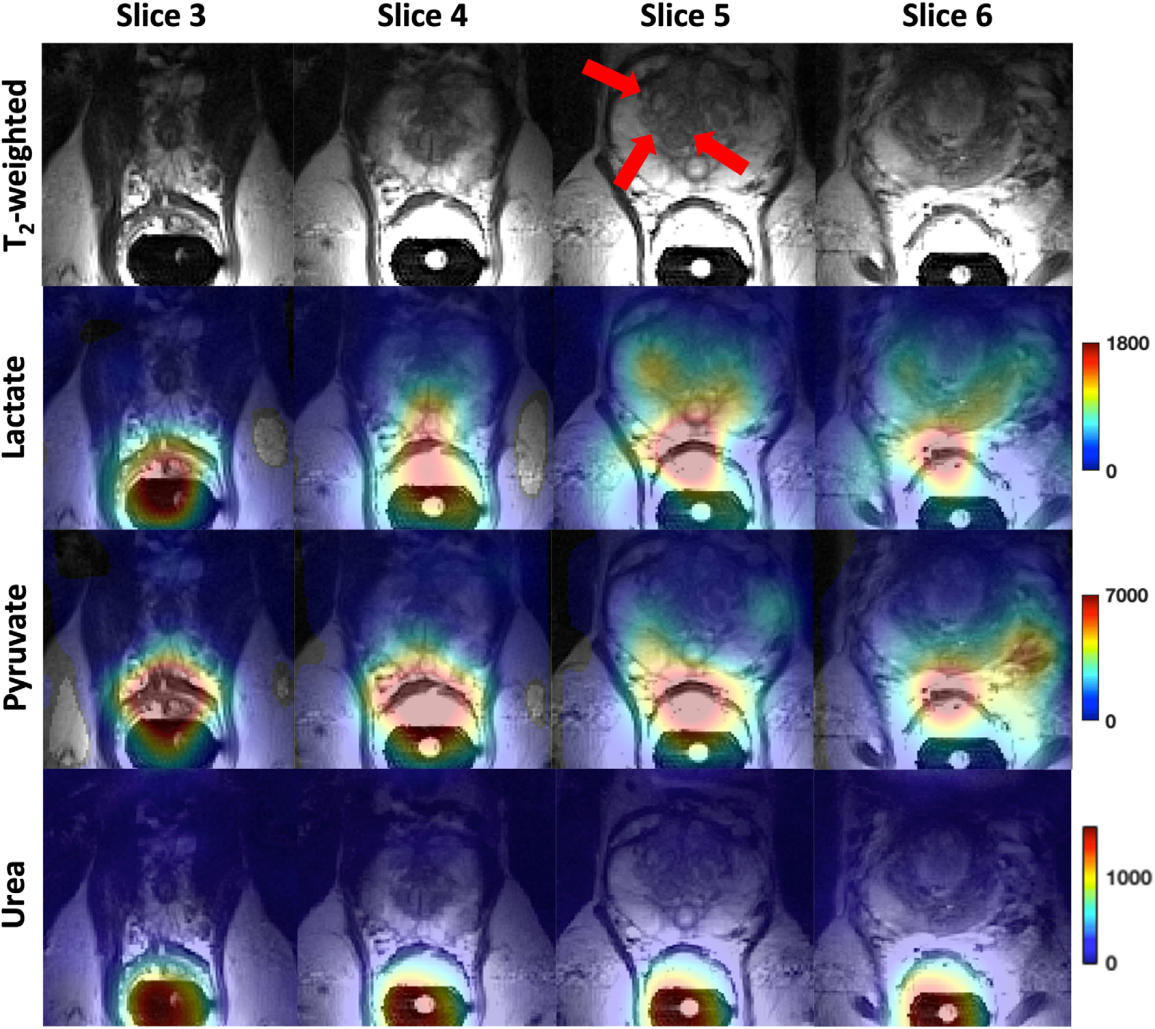
Axial slices of 3D metabolite maps for [1‐^13^C] lactate and [1‐^13^C] pyruvate signal intensities were overlaid onto T_2_W images obtained for the patient (subject 2) whose mpMRI was shown in Fig. 4. This measurement was taken 31 seconds post‐injection; each set of metabolite maps is scaled individually with spatial interpolation performed to improve visualization.

Figure 4 shows examples of mpMRI from a patient (subject 2) with a reported right‐sided, anterior, transition zone, biopsy‐confirmed Gleason 3 + 4 tumor. The tumor, as identified on targeted biopsy, is outlined with red arrows in each of the images. Figure 5 shows metabolite images for [1‐^13^C] pyruvate, [1‐^13^C] lactate, and [^13^C] urea (for spatial reference), derived from ME‐bSSFP echo data, overlaid on ^1^H T_2_W axial images from the same patient (subject 2). The images shown were interpolated using a cubic filter, for visualization purposes. Although all metabolites are shown with the same color map, they are scaled individually, as per the scale on the far‐right side of each set of metabolite images.

**FIGURE 6:**
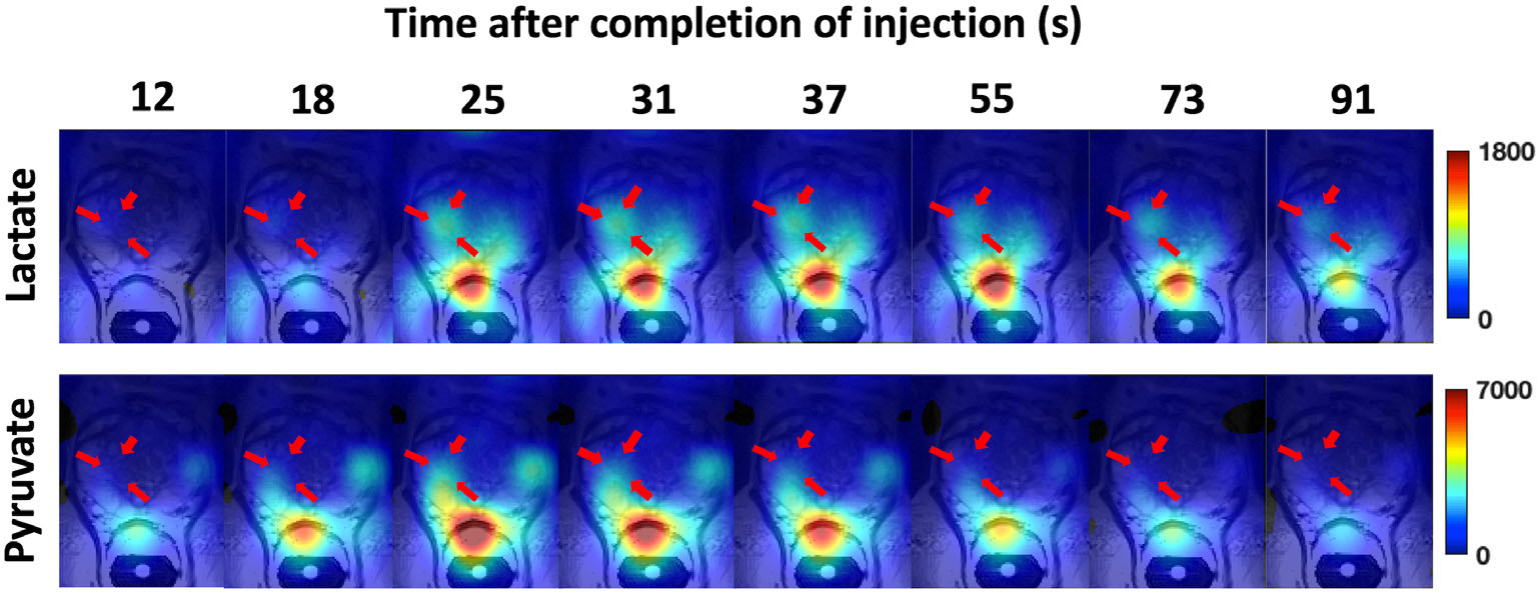
Signal intensity images for [1‐^13^C] pyruvate and [1‐^13^C] lactate (subject 2) were overlaid on a selected ^1^H T_2_W MR slice (slice 5 from Fig. 5) showing measurements up to 91 seconds post‐injection of hyperpolarized [1‐^13^C] pyruvate.

A further set of maps, show the dynamic change in signals from metabolites within the prostate (Fig. 6). The [1‐^13^C] pyruvate signal appeared to peak between 24 and 37 seconds after completion of the injection (peak SNR ~60), with homogeneous distribution over the entire prostate, except for a stronger signal close to the coil. The [1‐^13^C] lactate signal (peak SNR ~20) peaked between 31 and 43 seconds and showed spatial heterogeneity.

**FIGURE 7:**
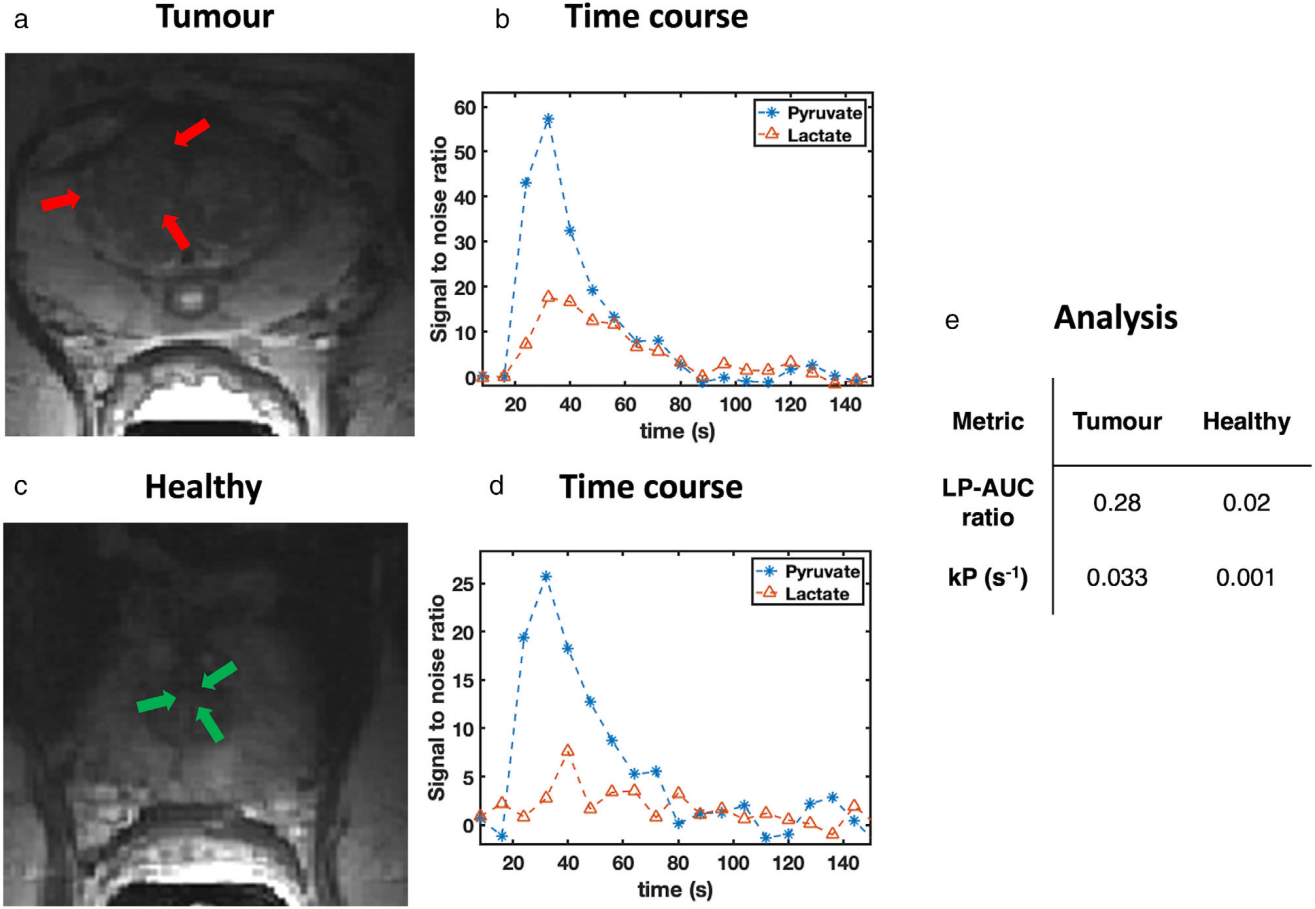
Signal‐to‐noise time courses for pyruvate (blue) and lactate (red) for the (a) tumor site (time course shown in b) and (c) a healthy tissue area (time course shown in d) for subject 2. The regions analyzed included (a) the tumor and (c) the healthy transition zone (TZ). The area‐under‐the‐curve (AUC) ratios calculated from the lactate to pyruvate time courses are shown (LP_AUC_ ratio), as well as the forward rate constant (*k*_P_) for a one directional kinetic model was also derived for both the healthy and tumorous regions. The metrics were then tabulated (e).

**FIGURE 8:**
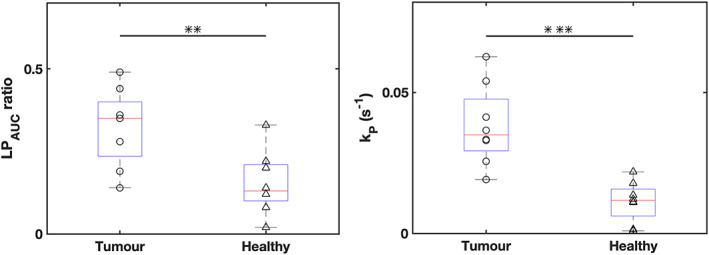
Differences in lactate to pyruvate area under the curve ratios (a) and forward rate constant, *k*_P_ (b), between healthy tissue and tumor ROIs. 95% confidence intervals are shown for each set in bold, while the results of a paired *t*‐test between tumor and healthy ROI values are also included in the subheading for each figure.

For the same patient, a higher degree of pyruvate metabolism (Fig. 7), as quantified using LP_AUC_ ratios and kinetic rate constants (*k*_P_) can be seen in tumor regions (Fig. 7b) when compared to healthy tissue (Fig. 7d). This was found to extend to all patients with the metrics extracted from tumor ROIs found to be statistically significantly higher than those in healthy regions (Table 2). This was demonstrated by the lack of overlap in the 95% confidence intervals between cancerous and healthy tissue metrics and with *P* values demonstrating significant differences between the two tissue types across both metrics (Fig. 8).

**TABLE 2:** The mean, SD, and 95% CIs for Lactate‐to‐Pyruvate AUC Ratios and *k*_P_ Constants in Biopsy Confirmed Tumors and Healthy Regions for All Subjects Involved in This Study

Subject	Tumor		Healthy	
	AUC	*k*_P_ (sec^−1^)	AUC	*k*_P_ (sec^−1^)
1	0.49	0.054	0.33	0.022
2	0.28	0033	0.02	0.001
3	0.19	0.033	0.12	0.011
4	0.14	0.025	0.08	0.012
5	0.35	0.063	0.20	0.014
6	0.35	0.019	0.12	0.001
**7**	0.44	0.041	0.22	0.018
8	0.36	0.037	0.14	0.011
Mean	0.33	0.038	0.15	0.011
SD	0.12	0.014	0.10	0.007
95% CIs	0.26	0.028	0.07	0.006
	0.40	0.048	0.23	0.016

AUC = area under the curve ratio; *k*_P_ = enzyme rate constant for the conversion of pyruvate into lactate.

## Discussion

This study demonstrated the successful implementation of ME‐bSSFP as a means of quantifying PCa metabolism using HP‐MRI. This study achieved its primary outcome by showing the conversion of [1‐^13^C] pyruvate into [1‐^13^C] lactate in real time and by quantifying the dynamic data through LP_AUC_ ratios derived from temporal metabolite signal curves and the enzyme rate constant, *k*_*P*_. Correlations were also observed between simulated and experimental ME‐bSSFP signals, fulfilling our secondary objective.

The efficiency of the broadband excitation suggested that there was no need for spectral‐spatial selective metabolite excitations, which may be susceptible to field inhomogeneities. Full volumetric prostate coverage seems necessary for HP‐MRI to be considered as a diagnostic tool for PCa and for evaluation of its clinical potential.

The use of the ME‐bSSFP MRI pulse sequence for HP‐MRI has previously been reported in preclinical studies.^12^, ^20^ Translation of the technique from preclinical to clinical scanners, however, necessitated further optimization. Here, we identified an optimal TR/FA combination with an in silico model, performing empirical studies to validate modeled data.^19^, ^24^ The Pearson correlation coefficients between empirical and simulated signals were in a similar range to a previous study using the same in silico model.^23^ While a high FA was found to afford an increased signal, consideration had to be given to limiting FA to protect the nonrenewable hyperpolarized longitudinal magnetization. SAR concerns were also considered. Lower FA (<30°) and the use of a limited number of dynamic acquisitions within a specified time were required to keep the nuclei independent, SAR within the acceptable range. This was balanced against having a too low FA as this would have resulted in decreased SNR. SNR was furthermore limited by the sensitivity profile of the endorectal coil, with sensitivity decreasing with distance from the coil.^30^ To maintain diagnostic quality imaging, ensure full prostate coverage, and achieve adequate temporal resolution, voxel dimensions for hyperpolarized imaging were set to 1.13 × 1.13 × 1 cm^3^ with a FOV of 90 × 90 × 80 mm^3^. The selected spatial resolution afforded a limited number of excitations for imaging, in turn allowing for the maintenance of an acceptable longitudinal hyperpolarized signal to allow for repeated temporal acquisitions. With these constraints, a limited range of acceptable TRs were identified as indicated by the in silico model, that is, 15.8 msec, 17.6 msec, and 21 msec. A TR of 17.6 msec, despite being at the edge of a pass band, was not chosen due to a compromised refocusing behavior during the transient phase of ME‐bSSFP.^25^ While both TRs, 15.8 msec and 21.0 msec, were suitable, the former was chosen to achieve a better temporal resolution. The total acquisition time for imaging the full prostate, including 6‐fold averaging, was 6 seconds.

We observed hyperpolarized pyruvate and lactate signals in all patients within this study (*n* = 8), both within prostate tissue and the rectal wall. These signals were corroborated by nonlocalized spectroscopic signals acquired during the same scanning sessions. Compared with external coils, an endorectal coil offers the advantage of higher sensitivity, which is critical for studies such as these that are very demanding of SNR.^6^, ^30^ However, their sensitivity profile is spatially dependent; it is highest in the vicinity of the coil and drops off with distance.^30^ For this reason, and because of its highly vascular nature, particularly strong lactate and pyruvate signals are seen from the rectal wall. This can result in substantial partial volume effects for nearby posterior tumors, as both may fall within the same voxel and complicate the visual display of metabolite maps. For visualization purposes, the image was cropped to display signals from just the prostate region. A higher spatial resolution would be beneficial; however, it should be noted that this would compromise temporal resolution and require a greater number of excitations and phase encoding steps, both of which are likely to negatively impact the longitudinal hyperpolarized signal.^25^

Analysis of signal curves within segmented ROIs demonstrated statistically significant differences in values for LP_AUC_ and *k*_P_ rate constants between healthy and biopsy‐confirmed tumor tissue, as seen in previous studies.^6^ Note that the kinetic model used in this study (SI6) is not fitting a pure T_1_‐decay but an apparent decay rate that is naturally the composition of T_1_ and T_2_ decays under effect of the pulse sequences excitations, as described in the literature.^25^ However, given the major contribution of T_1_ to the decay rate at the FA of 24: >95%*T_1_ and <5%*T_2_ (Ref 25, eq. 7), we assume a very small dependency to variations in T_2_ and thus approximate the modeled decay rate here as T_1_.

ME‐bSSFP has been demonstrated here as a feasible method by which to quantify PCa metabolism, and while the technique is now available for clinical evaluation, further parameter optimization and exploration of strategies such as compressed sensing can be pursued.^16^ As mentioned, the of the primary objectives for this study was to achieve full prostate coverage, as such our first compromise, due to the decaying nature of the hyperpolarized signal, involved selecting a 3D volume with lower spatial resolution, over a high‐resolution single‐slice acquisition. Due to the greater number of pulses introduced through having to excite multiple slices, which inevitably decay the hyperpolarized state faster, a larger voxel size was employed to maximize the potential signals acquired. The voxel size finally selected was chosen such that signals from the largest prostates, with potential tumors up to 60 mm away from the endorectal could be acquired. Finer resolutions may be possible, however, would require compromising full organ coverage.

A potential application of HP‐MRI involves monitoring disease burden, this, however, would require the identification of an appropriate biomarker by which to quantify HP‐MRI, several studies have attempted to do this.^28^, ^31^ Second, an understanding a numerical range of values of the aforementioned biomarker, in the context of disease progression would need to be established, much like SUV value in PET imaging, for HP‐MRI to provide value to clinical radiologists.^32^

## Limitations

Balanced SSFPs high signal efficiency, for example, with improved SNR over spoiled gradient echo (GRE) sequences, is generally concluded from spin dynamics in the steady state, where the generated magnetization converged toward an equilibrium between T_1_ and T_2_ relaxation and re‐excitation by RF. This assumption naturally is not valid for hyperpolarized magnetization, which is in a far nonequilibrium state. Application of bSSFP for HP‐MRI thus requires understanding the signal evolution during the transient phase of bSSFP, for example, described by Scheffler for the on‐resonant case, and its sensitivity to off‐resonances.^25^ Optimizing the TRs and FAs means iteratively adjustment of both parameters to the off‐resonances and the T_1_‐ and T_2_‐constants for all hyperpolarized metabolites. Furthermore, the exact off‐resonances as well as the T_1_ and T_2_‐constants of the HP metabolites are unknown for in vivo conditions and must be assumed. Two subsequent injections of HP metabolites into the same patient to measure first off‐resonance and T_1_/T_2_ and only then metabolite distribution and dynamics seems unfeasible at the current situation with the available limited hyperpolarization methods, for example, limitation of the SpinLab to maximum four HP batches, expenses for and current lack of clinical fluid paths, and so on.

In summary, the above arguments make bSSFP not a generally easy‐to‐apply, that is, plug‐and‐play, sequence for HP‐MRI, but requires distinct preparation for each application. We demonstrated that the above limitations of bSSFP for application in HP‐MRI on PCa can be overcome, by approximate assumptions about T_1_/T_2_ of [1‐^13^C] pyruvate and lactate and precise adjustment of the transmit and receive frequency using a ^13^C‐urea phantom.

Finally, the small cohort size in this study is not sufficient to make conclusions regarding biological trends in larger populations. It was, however, of sufficient size to demonstrate, as a proof‐of‐concept, the ME‐bSSFP sequence's propensity for metabolic prostate imaging, using HP‐MRI.

## Conclusion

This study demonstrated the optimization and application of a ME‐bSSFP sequence in the metabolic imaging of PCa in conjunction with hyperpolarized ^13^C‐MRI. We successfully demonstrated, with whole organ coverage, quantitative differentiation of metabolism in tumorous and healthy tissue in eight patients using the same ME‐bSSFP imaging protocol. The protocol was achieved from a numerical signal simulator, whose results were validated by empirical phantom measurements beforehand. With appropriate training of the staff and well‐designed standardized operation procedures, HP‐MRI in PCa is feasible using ME‐bSSFP.

## Supporting information


**Figure S1** field map slices generated via the processing of a dual gradient echo sequence, via Eq. 1, for subject 1.
**Figure S2**: Segmented abdominal field maps produced via drawing a region of interest around the data seen in Figure S2. This allows for a localized understanding of field inhomogeneity.
**Figure S3**: Variation in field inhomogeneity in the prostate, across all patients, shown in the form of a boxplot (left) and a histogram (right).
**Figure S4**: (a) Chemical reactions that [1‐^13^C]‐pyruvate undergoes in this series of experiments. Pyruvate hydrate and pyruvate are in a pH‐dependent equilibrium, while pyruvate is then enzymatically converted into lactate via lactate dehydrogenase. Importantly, the rate of lactate generation is several orders of magnitude greater than the production of pyruvate hydrate. The consumption of pyruvate induces an equilibrium imbalance resulting in the formation of pyruvate from pyruvate hydrate. (b) The differential equations, for the enzymatic conversion of pyruvate to lactate, which describe the forward (top) and reverse (bottom) reactions, respectively, in terms of rate constants *k*_P_ (forward) and *k*_L_ (reverse). (c) These equations were adapted to eliminate the reverse reaction in the fitting process by assuming *k*_L_ = 0 producing a one directional kinetic model from which *k*_P_ and the T_1_ of pyruvate were calculated. (d) The graphical methods used for analysis of the metabolite signal time curves in this study. The ratio of the lactate to pyruvate signals when lactate is at a maximum was calculated. The second graphical metric involved deriving the ratio of the area under the curve ratio of lactate to pyruvate.
**Figure S5**: Nonlocalized spectra obtained 24 s after the completion of injection of hyperpolarized [1‐^13^C] pyruvate. The vertical dotted lines show the frequencies used during the reconstruction of metabolite maps from the echo images.
**Figure S6**: Capability of ME‐bSSFP sequence in achieving full prostate coverage (subject 2). Axial slices of 3D signal intensity metabolite maps for [1‐^13^C] lactate and [1‐^13^C] pyruvate signal intensities were overlaid onto T_2_W images obtained for the patient (subject 2) whose mpMRI was shown in Figure 4 (main text). This measurement was taken 31 seconds postinjection; each set of metabolite maps is scaled individually with interpolation performed to improve visualization. These metabolite maps show how full prostate coverage is achieved after injection of hyperpolarized [1‐^13^C] pyruvate
**Figure S7**: Signal intensity images for [1‐^13^C] pyruvate and [1‐^13^C] lactate (subject 2) were overlaid on a selected ^1^H T_2_W MR slice (slice 5 from Figure 5—main text) showing measurements up to 91 seconds postinjection of hyperpolarized [1‐^13^C] pyruvate.
**Table S1**: (Above) Biopsy information for patients recruited to this study including time between most recent biopsy and HP‐MR scan, location of lesion and pathology results.
**Table S2**: (Below) Summary of hyperpolarized [1‐^13^C‐] pyruvate properties, prior to injection, as well as time taken to inject, for all patients involved in this study.

## References

[1] Cancer Research UK . Prostate cancer statistics [internet]. 2018 Available from: https://www.cancerresearchuk.org/health-professional/cancer-statistics/statistics-by-cancer-type/prostate-cancer#heading-Zero

[2] Johnson LM , Turkbey B , Figg WD , Choyke PL . Multiparametric MRI in prostate cancer management. Nat Rev Clin Oncol 2014;11:346‐353. 24840072 10.1038/nrclinonc.2014.69 PMC6330110

[3] Giganti F , Moore CM . A critical comparison of techniques for MRI‐targeted biopsy of the prostate. Transl Androl Urol 2017;6(3):432‐443. 28725585 10.21037/tau.2017.03.77 PMC5503959

[4] Frankel S , Smith GD , Donovan J , Neal D . Screening for prostate cancer. Lancet 2003;361:1122‐1128. 12672328 10.1016/S0140-6736(03)12890-5

[5] Jensen C , Carl J , Boesen L , Christian N , Østergaard LR . Assessment of prostate cancer prognostic Gleason grade group using zonal ‐ specific features extracted from biparametric MRI using a KNN classifier. J Appl Clin Med Phys 2019;20:146‐153. 30712281 10.1002/acm2.12542 PMC6370983

[6] Nelson SJ , Kurhanewicz J , Vigneron DB , et al. Metabolic imaging of patients with prostate cancer using hyperpolarized [ 1–13 C ] pyruvate. Sci Transl Med 2013;5(198):198ra108. 10.1126/scitranslmed.3006070 PMC4201045 23946197

[7] Levin YS , Albers MJ , Butler TN , Spielman D , Peehl DM , Kurhanewicz J . Methods for metabolic evaluation of prostate cancer cells using proton and (13)C HR‐MAS spectroscopy and [3–(13)C ] pyruvate as a metabolic substrate. Magn Reson Med 2009;1098:1091‐1098. 10.1002/mrm.22120 PMC2783184 19780158

[8] Siddiqui S , Kadlecek S , Pourfathi M , et al. The use of hyperpolarized carbon‐13 magnetic resonance for molecular imaging. Adv Drug Deliv Rev 2017;113:3‐23. 27599979 10.1016/j.addr.2016.08.011 PMC5783573

[9] Ardenkjær‐Larsen JH , Fridlund B , Gram A , et al. Increase in signal‐to‐noise ratio of >10,000 times in liquid‐state NMR. Proc Natl Acad Sci U S A 2003;100(18):10158‐10163. 12930897 10.1073/pnas.1733835100 PMC193532

[10] Golman K , In 't Zandt R , Thaning M . Real‐time metabolic imaging. Proc Natl Acad Sci U S A 2006;103(30):11270‐11275. 16837573 10.1073/pnas.0601319103 PMC1544077

[11] Abeyakoon O , Latifoltojar A , Gong F , et al. Hyperpolarised ^13^C MRI: A new horizon for non‐invasive diagnosis of aggressive breast cancer. BJR|Case Rep 2019;5:20190026. 31555479 10.1259/bjrcr.20190026 PMC6750630

[12] Milshteyn E , Larson PEZ , Von MC , Gordon JW , Zhu Z , Vigneron DB . High spatiotemporal resolution bSSFP imaging of hyperpolarized [1‐^13^C] pyruvate and [1‐^13^C] lactate with spectral suppression of alanine and pyruvate‐hydrate. Magn Reson Med 2018;80:1048‐1060. 29451329 10.1002/mrm.27104 PMC5980670

[13] Rodrigues TB , Serrao EM , Kennedy BWC , Hu D , Kettunen MI , Brindle KM . Magnetic resonance imaging of tumor glycolysis using using hyperpolarized 13C‐labeled glucose. Nat Med 2014;20(1):93‐98. 24317119 10.1038/nm.3416 PMC3886895

[14] Sriram R , Nguyen J , Santos JDL , et al. Molecular detection of inflammation in cell models using hyperpolarized 13 C‐pyruvate. Theranostics 2018;8(12):3400‐3407. 29930738 10.7150/thno.24322 PMC6010986

[15] Gordon JW , Chen H , Dwork N , Tang S , Larson PEZ . Fast imaging for hyperpolarized MR metabolic. J Magn Reson Imaging 2020;53(3):686‐702. 32039520 10.1002/jmri.27070 PMC7415658

[16] Chen H . Technique development of 3D dynamic CS‐EPSI for hyperpolarized 13C pyruvate MR molecular imaging of human prostate cancer. Magn Reson Med 2018;80(5):2062‐2072. 29575178 10.1002/mrm.27179 PMC6107425

[17] Gordon JW , Vigneron DB , Larson PEZ . Development of a symmetric Echo planar imaging framework for clinical translation of rapid dynamic hyperpolarized ^13^C Imaging. Magn Reson Med 2017;832:826‐832. 10.1002/mrm.26123 PMC4992668 26898849

[18] Dixon WT . Simple proton spectroscopic imaging. Radiology 1984;153(1):189‐194. 6089263 10.1148/radiology.153.1.6089263

[19] Leupold J , Månsson S , Stefan Petersson J , Hennig J , Wieben O . Fast multiecho balanced SSFP metabolite mapping of (1)H and hyperpolarized (13)C compounds. MAGMA 2009;22(4):251‐256. 19367422 10.1007/s10334-009-0169-z

[20] Peterson P . Fat quantification using multiecho sequences with bipolar gradients: Investigation of accuracy and noise performance. Magn Reson Med 2014;71(1):219‐229. 23412971 10.1002/mrm.24657

[21] Shang H , Sukumar S , von Morze C , et al. Spectrally selective three‐dimensional dynamic balanced steady‐state free precession for hyperpolarized C‐13 metabolic imaging with spectrally selective radiofrequency pulses. Magn Reson Med 2017;78(3):963‐975. 27770458 10.1002/mrm.26480 PMC5400740

[22] Wieben O , Leupold J . Multi‐echo balanced SSFP imaging for iterative Dixon reconstruction. Proc Intl Soc Mag Reson Med 2005;13:13‐2386.

[23] Müller CA , Braeuer M , Düwel S , et al. Dynamic 2D and 3D mapping of hyperpolarized pyruvate to lactate conversion in vivo with efficient multi‐echo balanced steady‐state free precession at 3 T. NMR Biomed 2020;33:e4291. 32154970 10.1002/nbm.4291

[24] Perman WH , Bhattacharya P , Leupold J , et al. Fast volumetric spatial‐spectral MR imaging of hyperpolarized C‐labeled compounds using multiple echo 3D bSSFP. Magn Reson Imaging [Internet] 2010;28(4):459‐465. 20171034 10.1016/j.mri.2009.12.003 PMC2860036

[25] Scheffler K . On the transient phase of balanced SSFP sequences. Magn Reson Med 2003;49(4):781‐783. 12652552 10.1002/mrm.10421

[26] Bieri O , Scheffler K . Fundamentals of balanced steady state free precession MRI. J Magn Reson Imaging 2013;38(1):2‐11. 23633246 10.1002/jmri.24163

[27] Leupold J , Wieben O , Månsson S , et al. Fast chemical shift mapping with multiecho balanced SSFP. Magn Reson Mater Physics, Biol Med 2006;19(5):267‐273. 10.1007/s10334-006-0056-9 17119904

[28] Daniels CJ , Mclean MA , Schulte RF , et al. A comparison of quantitative methods for clinical imaging with hyperpolarized 13C‐pyruvate. NMR Biomed 2016;29(4):387‐399. 27414749 10.1002/nbm.3468 PMC4833181

[29] Chowdhury R , Papoutsaki M‐V , Muller CA , Smith L . A reproducible dynamic phantom for sequence testing in hyperpolarised ^13^C‐magnetic resonance. Br J Radiol 2022;95(1134):20210770. 35230136 10.1259/bjr.20210770 PMC10996405

[30] Noworolski SM , Reed GD , Kurhanewicz J , Vigneron DB . Post‐processing correction of the endorectal coil reception effects in MR spectroscopic imaging of the prostate. J Magn Reson Imaging 2010;662:654‐662. 10.1002/jmri.22258 PMC2957824 20815064

[31] Larson PEZ , Chen H , Gordon JW , et al. Investigation of analysis methods for hyperpolarized 13C‐pyruvate metabolic MRI in prostate cancer patients. NMR Biomed 2018;31(11):e3997. 30230646 10.1002/nbm.3997 PMC6392436

[32] Lodge MA . Repeatability of SUV in oncologic 18F‐FDG PET. J Nucl Med 2020;58:523‐532. 10.2967/jnumed.116.186353 PMC5373499 28232605

