# Supplementary material for: Quantification of Prostate Cancer Metabolism Using 3D Multiecho bSSFP and Hyperpolarized [1‐13C] Pyruvate: Metabolism Differs Between Tumors of the Same Gleason Grade
Source: J Magn Reson Imaging. 2022 Oct 31;57(6):1865–75. doi: 10.1002/jmri.28467 (PMC10946772; doi:10.1002/jmri.28467)
Supplement: Supplementary file 1 — Figure S1 field map slices generated via the processing of a dual gradient echo sequence, via Eq. 1, for subject 1. Figure S2: Segmented abdominal field maps produced via drawing a region of interest around the data seen in Figure S2. This allows for a localized understanding of field inhomogeneity. Figure S3: Variation in field inhomogeneity in the prostate, across all patients, shown in the form of a boxplot (left) and a histogram (right). Figure S4: (a) Chemical reactions that [1‐13C]‐pyruvate undergoes in this series of experiments. Pyruvate hydrate and pyruvate are in a pH‐dependent equilibrium, while pyruvate is then enzymatically converted into lactate via lactate dehydrogenase. Importantly, the rate of lactate generation is several orders of magnitude greater than the production of pyruvate hydrate. The consumption of pyruvate induces an equilibrium imbalance resulting in the formation of pyruvate from pyruvate hydrate. (b) The differential equations, for the enzymatic conversion of pyruvate to lactate, which describe the forward (top) and reverse (bottom) reactions, respectively, in terms of rate constants k P (forward) and k L (reverse). (c) These equations were adapted to eliminate the reverse reaction in the fitting process by assuming k L = 0 producing a one directional kinetic model from which k P and the T1 of pyruvate were calculated. (d) The graphical methods used for analysis of the metabolite signal time curves in this study. The ratio of the lactate to pyruvate signals when lactate is at a maximum was calculated. The second graphical metric involved deriving the ratio of the area under the curve ratio of lactate to pyruvate. Figure S5: Nonlocalized spectra obtained 24 s after the completion of injection of hyperpolarized [1‐13C] pyruvate. The vertical dotted lines show the frequencies used during the reconstruction of metabolite maps from the echo images. Figure S6: Capability of ME‐bSSFP sequence in achieving full prostate coverage (subject 2). [file JMRI-57-1865-s001.docx]

**Supplementary information**

**SI1 - ME-bSSFP parameter optimisation**

**Simulation parameters**

The following parameters were kept fixed during the initial simulations: T_1_: 13.6 s, T_2_: – 0.6 s No. of pulses: 64, TE_center_: TR/2. Whilst combinations of the following parameters were used during the initial simulations: TR: 8-22 ms and FA – 5:35°. The initial simulations were performed at Δf: 0 Hz and Δf -385 Hz.

The following parameters were used during the simulations performed to identify an optimal TR: T_1_: 13.6 s, T_2_: – 0.6 s No. of pulses: 64, TE_center_: TR/2, Δf range: -700:100 Hz, FA: 24° and TR: 15.8/17.6/21.0 ms.

**Lactate phantom**

The lactate phantom used in this study prepared in a similar manner to that presented in a previous study (1). The T_1_ and T_2_ values measured for this phantom were 13.1 s and 0.6 s respectively. The volume of the cylindrical phantom was 2.3ml, with housing material machined from a polyetherimide stock. The phantom was also doped with sodium azide (0.23% w/v) and gadoteric acid (0.15% w/w).

**SI2 - B_0_ field inhomogeneities**

With prostate imaging involving the use of an endorectal coil, large inhomogeneities arise when air becomes trapped in the rectum, which may result in spectral shifts, potentially causing metabolites to appear in stop bands. As such wide and flat pass bands are preferred, when selecting a suitable TR. Dual gradient echo acquisitions were obtained for each subject after the completion of the me-bSSFP scans, from which field maps were calculated. This was done to better understand whether field inhomogeneity would influence signal amplitude from the selected ME-bSSFP parameters. The phase difference between each set of images was divided by the difference in echo time to afford a field map (Eq. S1):

|  | $\psi= \frac{\Delta\theta}{\Delta TE}$ | (S1) |
| --- | --- | --- |

Where ψ is the B_0_ inhomogeneity in Hz and Δθ is the difference in the phase images from the two echoes. An example of such a field map is shown below (Figure S1):


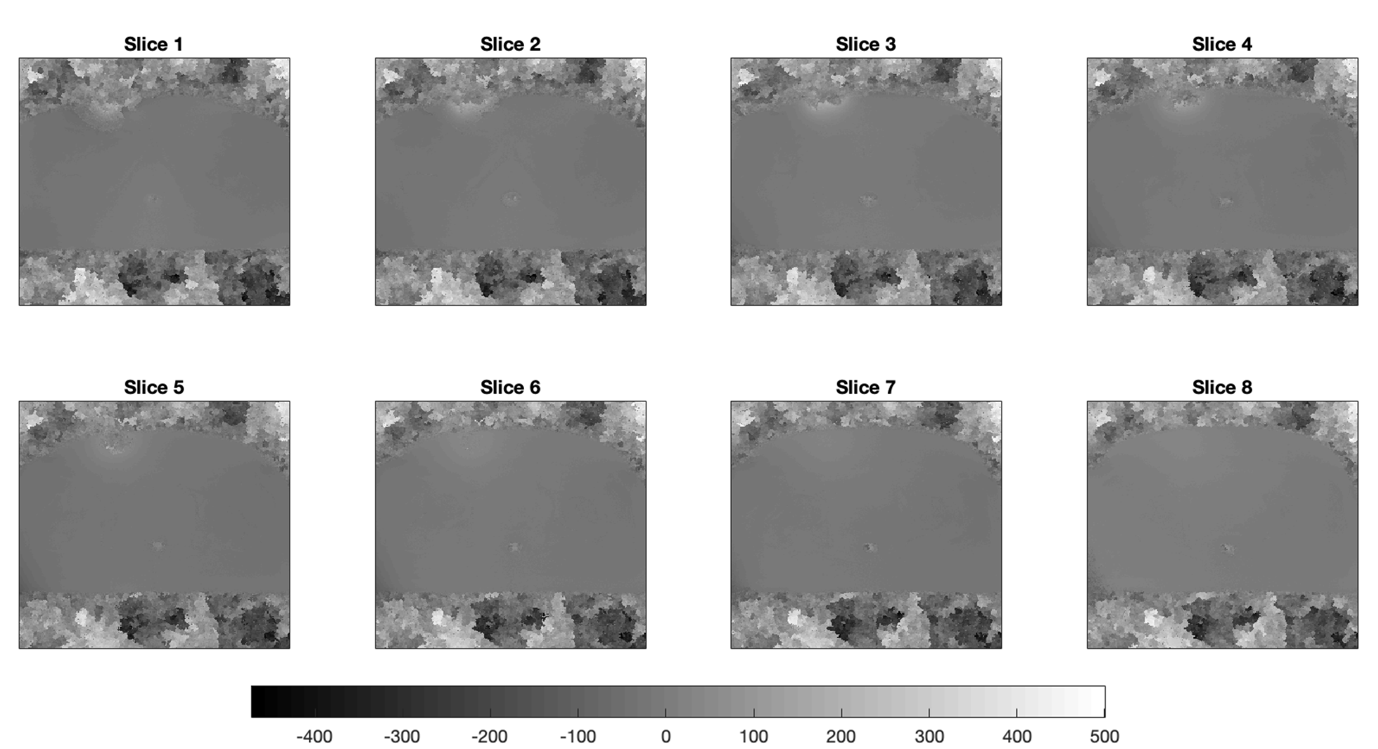


Figure S 1 - field map slices generated via the processing of a dual gradient echo sequence, via Eq.1, for Subject 1.

By segmenting the abdomen, the field inhomogeneities can be better visualised (Figure S2):


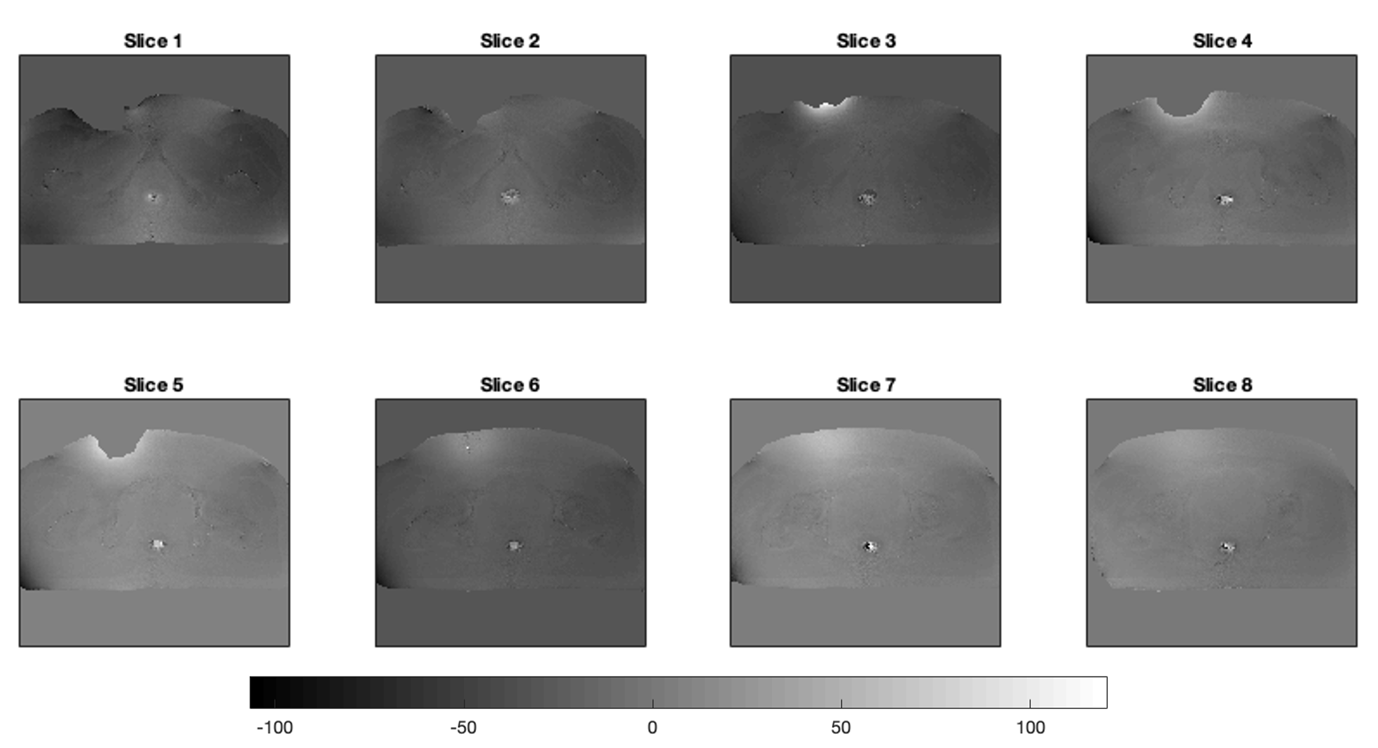


Figure S 2 - Segmented abdominal field maps produced via drawing a region of interest around the data seen in Figure 2. This allows for a localised understanding of field inhomogeneity.

Quantitative analysis of these field maps show a high signal in the rectum, which can be attributed to the presence of air. The variation across the entire abdomen, excluding the rectum is ±22Hz. the inhomogeneity in the endorectal coil itself, varies between -167 to 240Hz, across all patients recruited for this study and is in a range likely to be noise. Further analysis of the field maps showed that in the prostate there was a field inhomogeneity of 0.47±0.55 Hz (mean ± 95% CI), across the entire cohort (Figure S3).


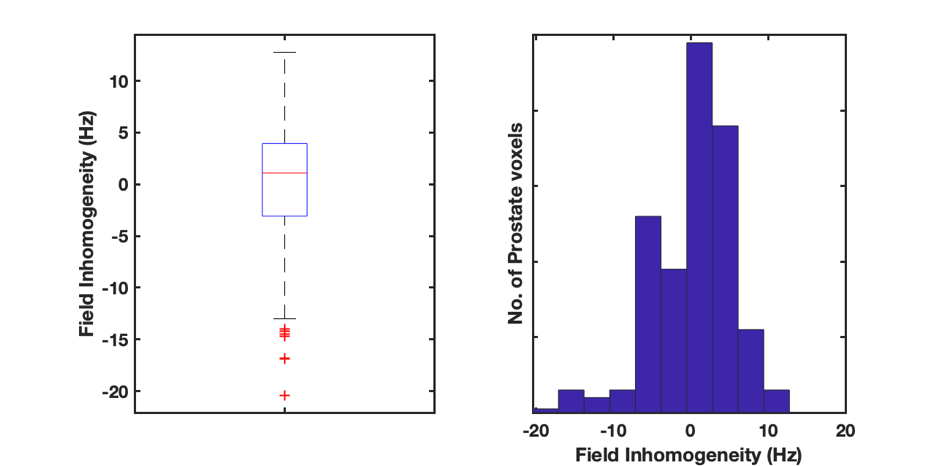


Figure S 3 - Variation in field inhomogeneity in the prostate, across all patients, shown in the form of a boxplot (left) and a histogram (right).

Field map analysis, alongside the earlier signal modelling was used to select an optimal TR. Despite a TR of 21.0ms showing both lactate and pyruvate appearing in the middle of pass bands a TR of 15.8ms was chosen due to the wider nature of the pass bands considering the potential effects of field inhomogeneity across the abdomen, excluding the rectum (Figure 3). The higher estimated M_T_ in said bands was also an influencing factor, whilst the shorter TR would contribute to a preferred, faster temporal resolution. Urea was not considered further when selecting TR and FA as it is not of metabolic interest, appearing only in a phantom within the endorectal coil, used for FA calibration, scanner frequency centring and physical positioning purposes.

**SI3 – IDEAL separation and metabolite frequencies**

Pyruvate and lactate maps were created from bipolar multi-echo (ME) data using iterative decomposition with echo asymmetries and least squares estimation (IDEAL). The theory and algorithm are originally described in *Reeder et al. 2004* with additional correction for bipolar echo effects as described in *Peterson 2013* and adapted for the separation of ^13^C metabolites in our application (2,3). On a pixel-by-pixel basis, the following signal model was assumed throughout all exams:

$\boldsymbol{S}=\boldsymbol{E}\cdot\boldsymbol{D}\cdot\boldsymbol{A}\cdot\boldsymbol{P}$

$\boldsymbol{S}_{7\times1}= \left[ \begin{aligned} S\left( t_{1} \right) \\ \vdots\\ S\left( t_{7} \right) \end{aligned} \right]$, $\boldsymbol{A}_{7\times4}=\left[ \begin{matrix} e^{i2\pi\Delta f_{L}t_{1}} & e^{i2\pi\Delta f_{PH}t_{1}} & e^{i2\pi\Delta f_{P}t_{1}} & e^{i2\pi\Delta f_{U}t_{1}} \\ e^{i2\pi\Delta f_{L}t_{2}} & e^{i2\pi\Delta f_{PH}t_{2}} & e^{i2\pi\Delta f_{P}t_{2}} & e^{i2\pi\Delta f_{U}t_{2}} \\ \vdots& \vdots& \vdots& \vdots\\ e^{i2\pi\Delta f_{L}t_{7}} & 0e^{i2\pi\Delta f_{PH}t_{7}} & e^{i2\pi\Delta f_{P}t_{7}} & e^{i2\pi\Delta f_{U}t_{7}} \end{matrix} \right]$,

$\boldsymbol{E}_{7\times7}=\left[ \begin{matrix} e^{\left( -1 \right)^{1}i\theta} & 0 & \cdots& 0 \\ 0 & e^{\left( -1 \right)^{2}i\theta} & \cdots& 0 \\ \vdots& \vdots& \ddots& 0 \\ 0 & 0 & 0 & e^{\left( -1 \right)^{7}i\theta} \end{matrix} \right]$, $\boldsymbol{D}_{7\times7}=\left[ \begin{matrix} e^{i2\pi\psi_{0}t_{1}} & 0 & \cdots& 0 \\ 0 & e^{i2\pi\psi_{0}t_{2}} & \cdots& 0 \\ \vdots& \vdots& \ddots& 0 \\ 0 & 0 & 0 & e^{i2\pi\psi_{0}t_{7}} \end{matrix} \right]$,

$\boldsymbol{P}_{4\times1}= \left[ \begin{aligned} \rho_{L} \\ \rho_{PH} \\ \rho_{P} \\ \rho_{U} \end{aligned} \right]$,

or written as a signal model equation for $t_{n}$:

$$S\left( t_{n} \right)= \sum_{m\in\{L,PH,P,U\}} (\rho_{m}\cdot e^{-i2\pi\left( \Delta f_{m}+\psi_{0} \right)t_{n}})\cdot e^{\left( -1 \right)^{n}i\theta}$$

with $S\left( t_{n} \right)$ being the signal recorded at echo time $t_{n}$, $\Delta f_{m}$ being the relative frequency of metabolite $m$ ($L$: Lactate, $PH$: Pyruvate-hydrate, $P$: Pyruvate, $U$: Urea), $\theta$ being the complex error for correction of the bipolarity effects, and $\psi_{0}$ the static offset of the magnetic field $B_{0}$ in Hertz. The values for $\Delta f_{m}$ were extracted from the interleaved spectral acquisitions using an automated peak search method (findpeaks(), MATLAB, R2022b) for each individual patient (Figure S1). The initial value for $\theta$ was set to zero and for $\psi_{0}$ to the corresponding pixel-value from the acquired B_0_ field map. The field map was therefore down sampled from the ^1^H- to the ^13^C-spatial resolution and converted into ^13^C frequencies by multiplication with $\gamma\left( {}^{13}C \right)/\gamma({}^{1}H)$. The whole maps for $\theta$ and $\psi_{0}$ were iteratively estimated using the algorithm described in *Reeder et al. 2004* for 15 iterations (2). The final estimation of the metabolite intensities $\rho_{m}$ was performed using the pseudo-inverse and inverse matrices for $\boldsymbol{A}$, $\boldsymbol{D}$, and $\boldsymbol{E}$.

$\boldsymbol{P}=\left( \boldsymbol{A}^{\boldsymbol{T}}\boldsymbol{A} \right)^{\boldsymbol{-1}}\boldsymbol{A}^{\boldsymbol{T}}\cdot\left( \boldsymbol{DE} \right)^{-1}\cdot\boldsymbol{S}$

The ME-bSSFP sequence used in the study utilised bipolar gradients. A bipolar gradient records the signal during an alternating trajectory through k-space. For technical implementations, the rows of ADC samples from even echoes thus are reverted or tagged with a reversion flag before being stored in a raw data matrix. In our case, the central ADC sample in odd echoes is at index five and in even echoes at index four (total number of ADC samples per echo: 8). To match the k-space centers in odd and even echoes the even echo signals were shifted by one discrete position. However, manually shifting the k-space data in a matrix and applying FFT2 results in complex image data with a linear phase roll in direction of the shift over the entire FOV. The magnitude image is unaffected (no visible artefacts). In order to correct for the phase roll in bipolar echo images the IDEAL model assumes an additional factor $e^{\left( -1 \right)^{n}\theta}$ with theta fitting a constant phase offset at the current image position that will alternate in even or odd echoes.

MATLAB scripts and functions for ^1^H B_0_ field map reconstruction, HP ^13^C MRI reconstruction (Siemens MRI data only), and IDEAL metabolite separation were version controlled and can be shared by the authors on request.

**SI4 – Bipolar gradients and correction**

A bipolar gradient records the signal during an alternating trajectory through k-space. For technical implementations, the rows of ADC samples from even echoes thus are reverted or tagged with a reversion flag before being stored in a raw data matrix. In our case, the central ADC sample in odd echoes is at index five and in even echoes at index four (total number of ADC samples per echo: 8). To match the k-space centers in odd and even echoes the even echo signals were shifted by one discrete position.

However, manually shifting the k-space data in a matrix and applying FFT2 results in complex image data with a linear phase roll in direction of the shift over the entire FOV. The magnitude image is unaffected (no visible artefacts). In order to correct for the phase roll in bipolar echo images the IDEAL model assumes an additional factor $e^{\left( -1 \right)^{n}\theta}$ with theta fitting a constant phase offset at the current image position that will alternate in even or odd echoes.

**SI5 – Power calibration**

Power calibration of the flip angle was performed using the [^13^C] urea phantom found within the endorectal used in this study. This involved varying the magnitude of the B1 field applied by the clamshell transmit coil in this study, which is in turn determined by the output voltage. A range of voltages are swept through, typically affording a sinusoidal signal response from the [^13^C] urea phantom, with increasing voltage. We used this procedure to determine the RF output necessary to achieve a 90° pulse. This was performed due to the variable loading of the transmitter coil occurring between different patients, changing the required transmit coil output. We found a reference voltage of 450W necessary to produce a 90° pulse for 1ms, using our transmit coil.

**SI6 - Data processing methods**


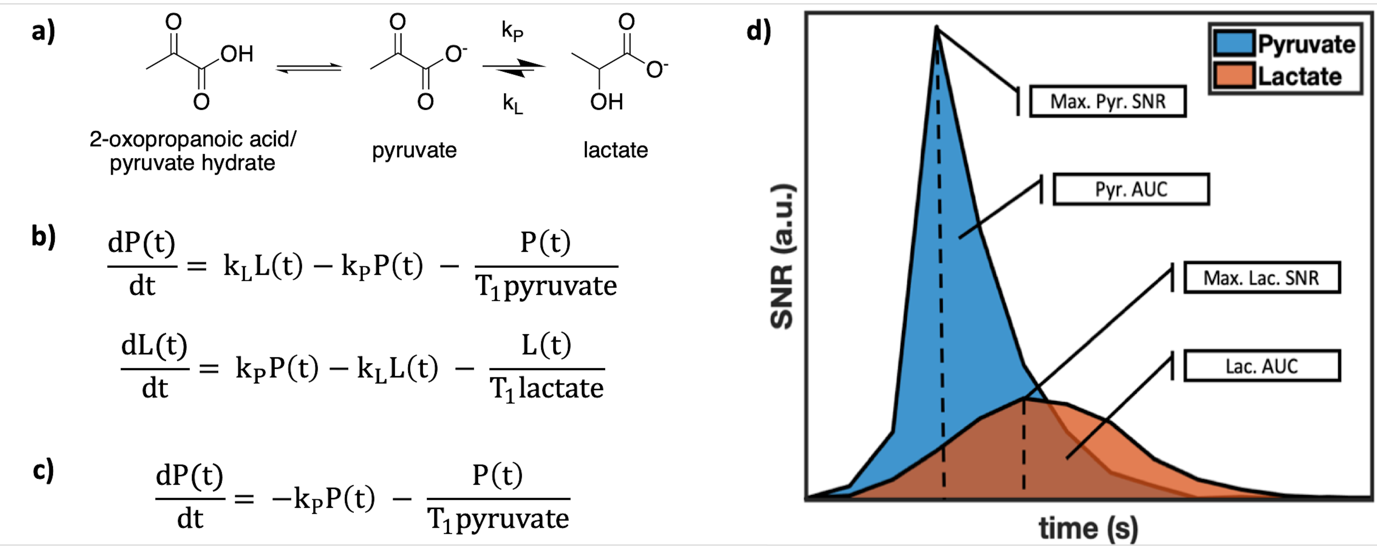


Figure S 4 - a) Chemical reactions that [1-^13^C]-pyruvate undergoes in this series of experiments. Pyruvate hydrate and pyruvate are in a pH dependent equilibrium, whilst pyruvate is then enzymatically converted into lactate via lactate dehydrogenase. Importantly, the rate of lactate generation is several orders of magnitude greater than the production of pyruvate hydrate. The consumption of pyruvate induces an equilibrium imbalance resulting in the formation of pyruvate from pyruvate hydrate. b) The differential equations, for the enzymatic conversion of pyruvate to lactate, which describe the forward (top) and reverse (bottom) reactions respectively in terms of rate constants k_P_ (forward) and k_L_ (reverse). c) These equations were adapted to eliminate the reverse reaction in the fitting process by assuming k_L_ = 0 producing a one directional kinetic model from which k_P_ and the T_1_ of pyruvate were calculated. d) The graphical methods used for analysis of the metabolite signal time curves in this study. The ratio of the lactate to pyruvate signals when lactate is at a maximum was calculated. The second graphical metric involved deriving the ratio of the area un the curve ratio of lactate to pyruvate.

Lactate to pyruvate area under the temporal curve (AUC) ratios (Figure S4d) were calculated as previously described (4). The one directional kinetic model (Figure S4c) used in this study was based on a set of coupled differential equations (Figure 4b) which describe the interconversion of pyruvate and lactate by the lactate dehydrogenase (LDH) enzyme. For our purposes it was assumed that the reverse reaction was negligible (k_L_ = 0). Using the above (Figure S4c) the rate of pyruvate to lactate conversion (kP) and pyruvate T_1_ was estimated.

**SI7 - Patient background**


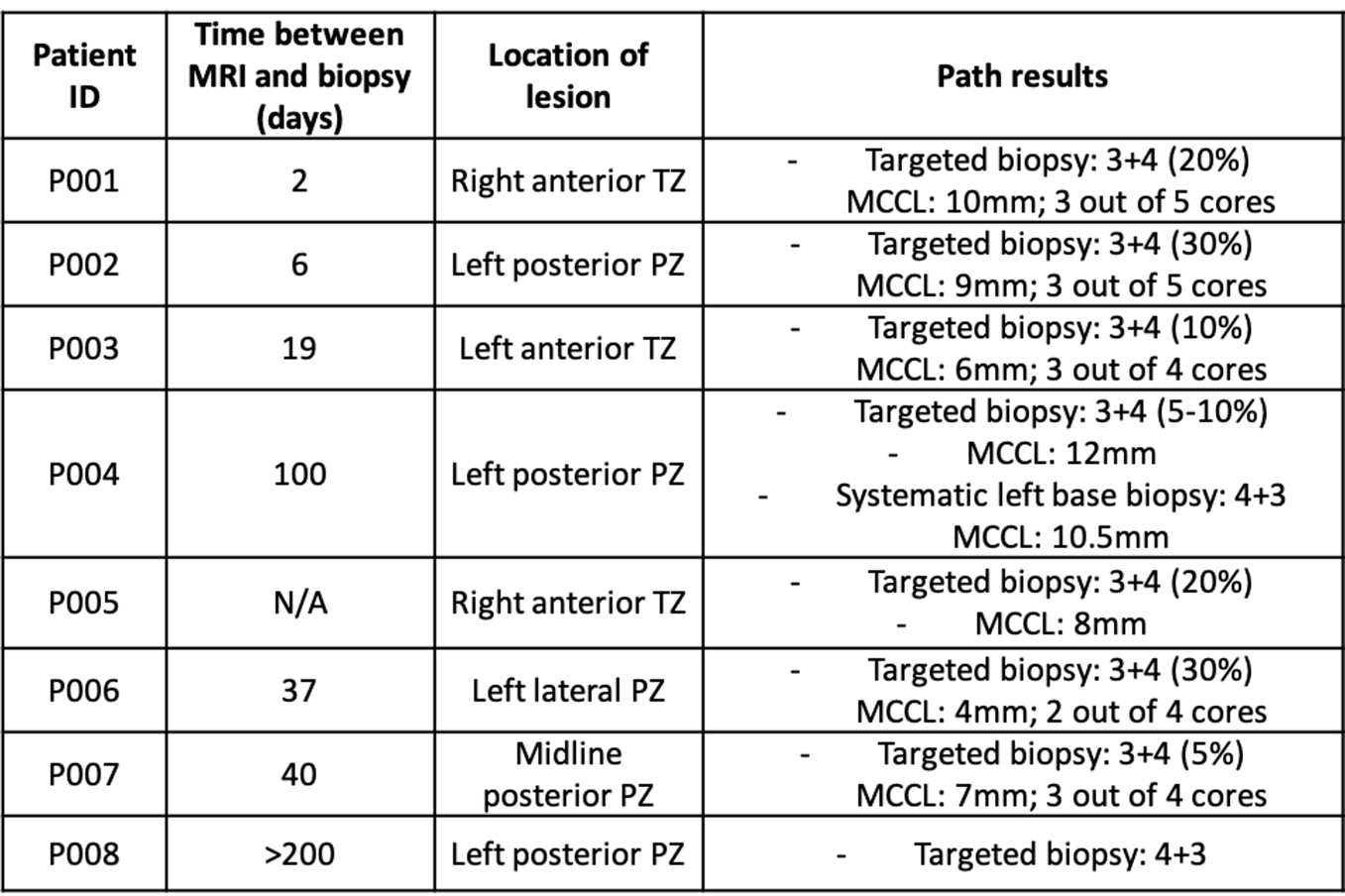


Table S1 – (Above) Biopsy information for patients recruited to this study including time between most recent biopsy and HP-MR scan, location of lesion and pathology results.

**SI8 - Fluid path results**


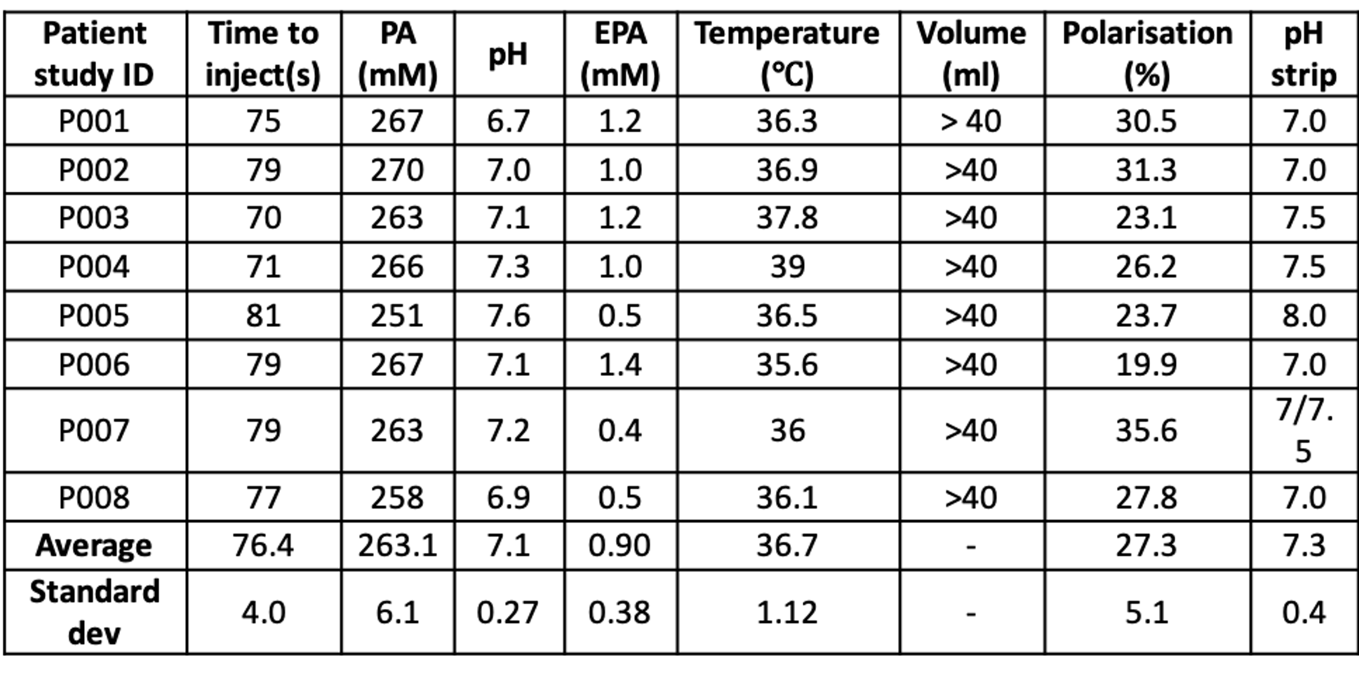
 *Table S2 – (Below) Summary of hyperpolarised [1-^13^C-] pyruvate properties, prior to injection, as well as time taken to inject, for all patients involved in this study.*

**SI9 – ^13^C-MR results**

**^13^C-MR Non-localised spectroscopy**


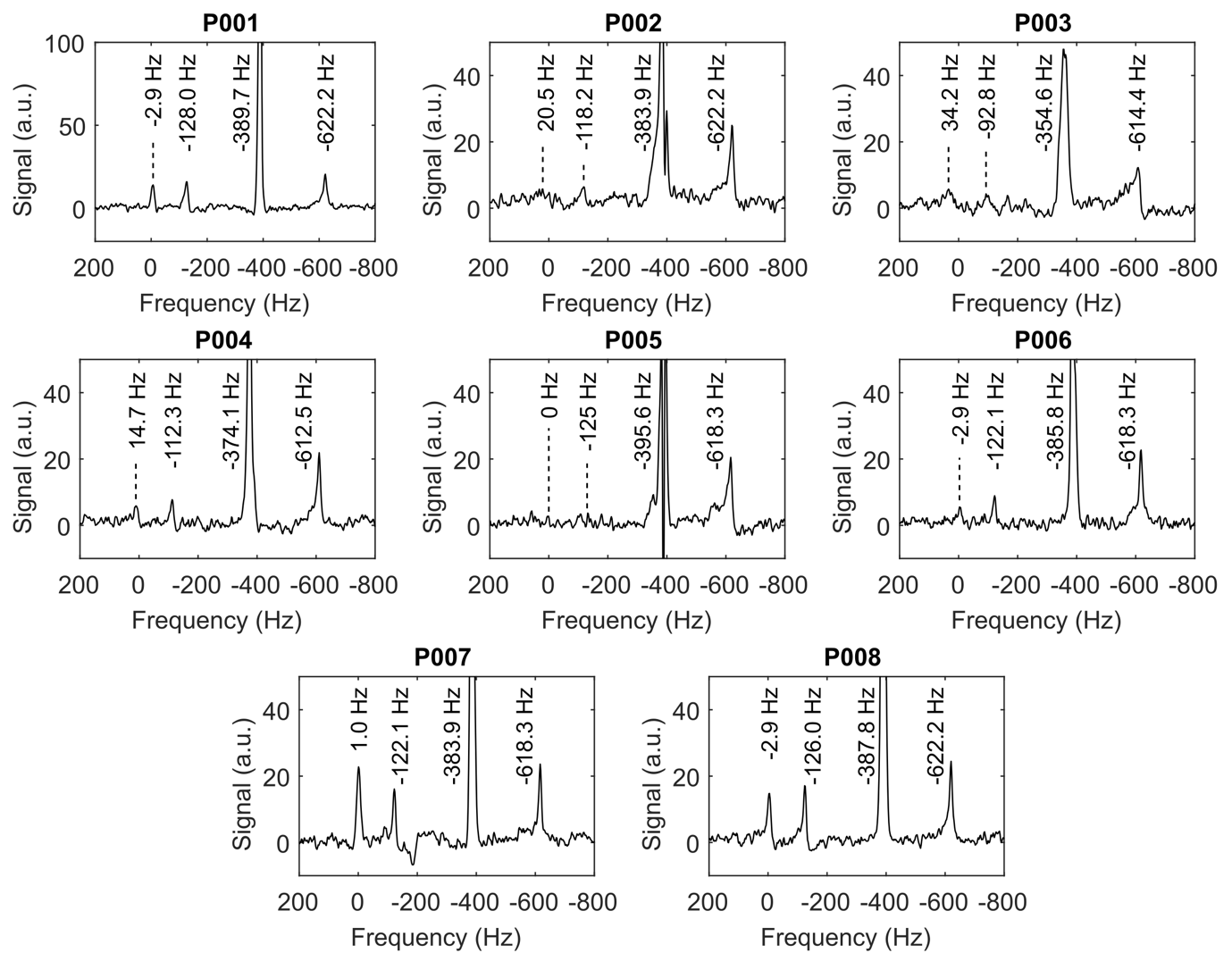


Figure S 5 – Non-localised spectra obtained 24s after the completion of injection of hyperpolarised [1-^13^C] pyruvate. The vertical dotted lines show the frequencies used during the reconstruction of metabolite maps from the echo images.

**^13^C-MR Imaging**


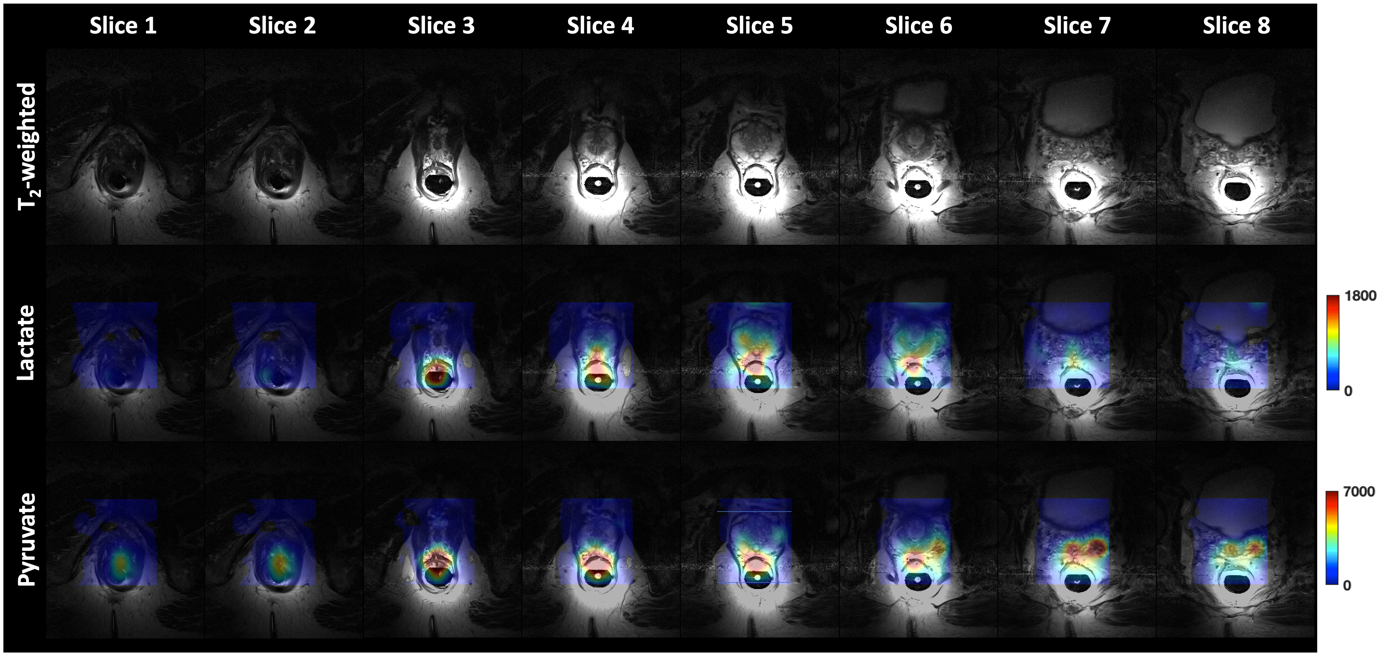


Figure S 6 - Capability of ME-bSSFP sequence in achieving full prostate coverage (subject 2). Axial slices of 3D signal intensity metabolite maps for [1-^13^C] lactate and [1-^13^C] pyruvate signal intensities were overlaid onto T_2_W images obtained for the patient (Subject 2) whose mpMRI was shown in Figure 4 (main text). This measurement was taken 31s post-injection; each set of metabolite maps is scaled individually with interpolation performed to improve visualisation. These metabolite maps show how full prostate coverage is achieved after injection of hyperpolarised[1-^13^C] pyruvate


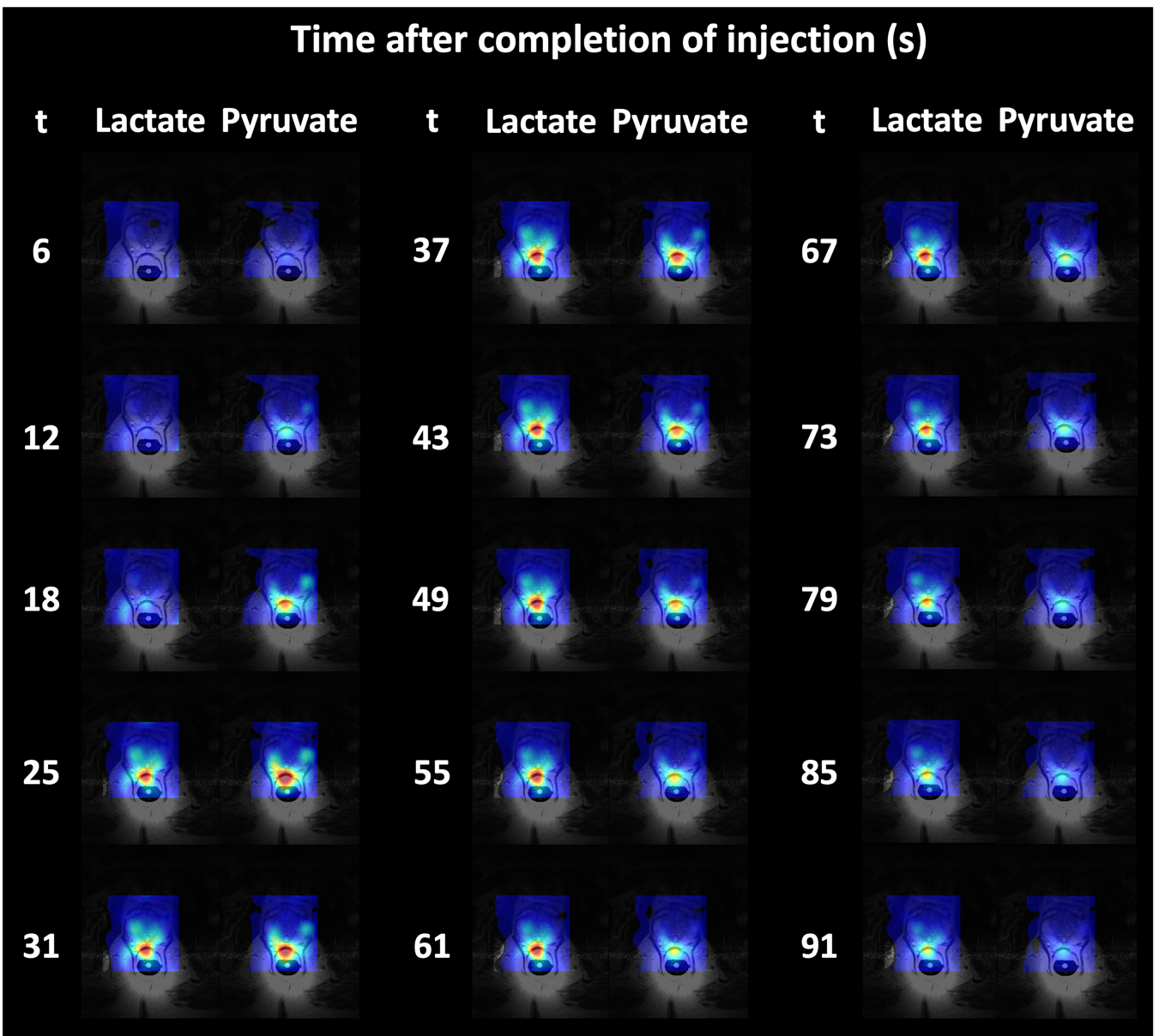


Figure S 7 - Signal intensity images for [1-^13^C] pyruvate and [1-^13^C] lactate (Subject 2) were overlaid on a selected ^1^H T_2_W MR slice (slice 5 from Figure 5 – main text) showing measurements up to 91s post-injection of hyperpolarised [1-^13^C] pyruvate.

**References**

1. Müller CA, Braeuer M, Düwel S, Skinner JG, Berner S, Leupold J, et al. Dynamic 2D and 3D mapping of hyperpolarized pyruvate to lactate conversion in vivo with efficient multi-echo balanced steady-state free precession at 3 T. 2020;(February):1–16.

2. Reeder SB, Wen Z, Yu H, Pineda AR, Gold GE, Markl M, et al. Multicoil Dixon Chemical Species Separation with an Iterative Least-Squares Estimation Method. Magn Reson Med. 2004;51(1):35–45.

3. Peterson P. Fat quantification using multiecho sequences with bipolar gradients: investigation of accuracy and noise performance. Magn Reson Med. 2014;71(1):219–29.

4. Hill DK, Orton MR, Mariotti E, Boult JKR, Panek R, Jafar M, et al. Model Free Approach to Kinetic Analysis of Real-Time Hyperpolarized 13 C Magnetic Resonance Spectroscopy Data. 2013;8(9):1–9.
